# Protein intake and type 2 diabetes mellitus: an umbrella review of systematic reviews for the evidence-based guideline for protein intake of the German Nutrition Society

**DOI:** 10.1007/s00394-023-03234-5

**Published:** 2023-09-17

**Authors:** Matthias B. Schulze, Julia Haardt, Anna M. Amini, Nicole Kalotai, Andreas Lehmann, Annemarie Schmidt, Anette E. Buyken, Sarah Egert, Sabine Ellinger, Anja Kroke, Tilman Kühn, Sandrine Louis, Katharina Nimptsch, Lukas Schwingshackl, Roswitha Siener, Armin Zittermann, Bernhard Watzl, Stefan Lorkowski, on behalf of the German Nutrition Society

**Affiliations:** 1 Department of Molecular Epidemiology, German Institute of Human Nutrition Potsdam-Rehbruecke, Arthur-Scheunert-Allee 114-116, 14558 Nuthetal, Germany; 2 Institute of Nutritional Science, University of Potsdam, Potsdam, Germany; 3 German Center for Diabetes Research (DZD), Munich-Neuherberg, Germany; 4 German Nutrition Society, Bonn, Germany; 5 Institute of Nutrition, Consumption and Health; Faculty of Natural Sciences, Paderborn University, Paderborn, Germany; 6 Department of Nutrition and Food Science, Nutritional Physiology, University of Bonn, Bonn, Germany; 7 Department of Nutrition and Food Science, Human Nutrition, University of Bonn, Bonn, Germany; 8 Department of Nutritional, Food and Consumer Sciences, Fulda University of Applied Sciences, Fulda, Germany; 9 The Institute for Global Food Security, Queen’s University Belfast, Belfast, Northern Ireland UK; 10 Faculty of Medicine and University Hospital, Heidelberg Institute of Global Health (HIGH), Heidelberg, Germany; 11 Department of Nutritional Sciences, University of Vienna, Vienna, Austria; 12 Center for Public Health, Medical University of Vienna, Vienna, Austria; 13 Department of Physiology and Biochemistry of Nutrition, Max Rubner-Institut, Karlsruhe, Germany; 14 Molecular Epidemiology Research Group, Max Delbrück Center for Molecular Medicine (MDC) in the Helmholtz Association, Berlin, Germany; 15 Institute for Evidence in Medicine, Faculty of Medicine, Medical Center-University of Freiburg, University of Freiburg, Freiburg, Germany; 16 Department of Urology, University Stone Center, University Hospital Bonn, Bonn, Germany; 17 Clinic for Thoracic and Cardiovascular Surgery, Herz- und Diabeteszentrum Nordrhein-Westfalen, Ruhr University Bochum, Bad Oeynhausen, Germany; 18 Institute of Nutritional Sciences, Friedrich Schiller University Jena, Jena, Germany; 19 Competence Cluster for Nutrition and Cardiovascular Health (nutriCARD), Halle-Jena-Leipzig, Germany

**Keywords:** Protein, Umbrella review, Guideline, Type 2 diabetes mellitus

## Abstract

**Purpose:**

Protein-rich foods show heterogeneous associations with the risk of type 2 diabetes (T2D) and it remains unclear whether habitual protein intake is related to T2D risk. We carried out an umbrella review of systematic reviews (SR) of randomised trials and/or cohort studies on protein intake in relation to risks of T2D.

**Methods:**

Following a pre-specified protocol (PROSPERO: CRD42018082395), we retrieved SRs on protein intake and T2D risk published between July 1st 2009 and May 22nd 2022, and assessed the methodological quality and outcome-specific certainty of the evidence using a modified version of AMSTAR 2 and NutriGrade, respectively. The overall certainty of evidence was rated according to predefined criteria.

**Results:**

Eight SRs were identified of which six contained meta-analyses. The majority of SRs on total protein intake had moderate or high methodological quality and moderate outcome-specific certainty of evidence according to NutriGrade, however, the latter was low for the majority of SRs on animal and plant protein. Six of the eight SRs reported risk increases with both total and animal protein. According to one SR, total protein intake in studies was ~ 21 energy percentage (%E) in the highest intake category and 15%E in the lowest intake category. Relative Risks comparing high versus low intake in most recent SRs ranged from 1.09 (two SRs, 95% CIs 1.02–1.15 and 1.06–1.13) to 1.11 (1.05–1.16) for total protein (between 8 and 12 cohort studies included) and from 1.13 (1.08–1.19) to 1.19 (two SRs, 1.11–1.28 and 1.11–1.28) (8–9 cohort studies) for animal protein. However, SRs on RCTs examining major glycaemic traits (HbA_1c_, fasting glucose, fasting insulin) do not support a clear biological link with T2D risk. For plant protein, some recent SRs pointed towards risk decreases and non-linear associations, however, the majority did not support an association with T2D risk.

**Conclusion:**

Higher total protein intake was possibly associated with higher T2D risk, while there is insufficient evidence for a risk increase with higher intakes of animal protein and a risk decrease with plant protein intake. Given that most SRs on plant protein did not indicate an association, there is possibly a lack of an effect.

**Supplementary Information:**

The online version contains supplementary material available at 10.1007/s00394-023-03234-5.

## Introduction

Globally, 537 million adults were living with type 2 diabetes mellitus (T2D) in 2021, and the prevalence is projected to rise to over 780 million by 2045 [1]. T2D, which accounts for the vast majority of diabetes cases, appears to be largely preventable by a balanced lifestyle. Besides obesity, a major modifiable risk factor, a suboptimal diet is considered a major contributor to the development of T2D. Evidence accumulated from both prospective observational studies and randomised controlled trials (RCTs) highlights the importance of single dietary factors in this context. Consumption of processed and unprocessed red meats as well as sugar-sweetened beverages have been observed being related to an increased T2D incidence, while whole grains, dairy products, nuts, green-leafy vegetables and coffee may reduce the risk of T2D [2–5]. Furthermore, the quality of carbohydrates (fibre, glycaemic index) and fats (saturated versus unsaturated fatty acids) as well as individual nutrients (iron, magnesium) have been identified to affect the risk of T2D [2, 6]. However, the role of total carbohydrate (CHO) and total fat intake, major macronutrients besides protein, appears to be less pronounced. For example, both prospective cohort studies as well as results from the Women’s Health Initiative RCT suggest that a general increase in CHO intake at the expense of fat is unlikely to affect T2D risk [7]. Protein-rich foods show inconsistent associations with the risk of T2D, with red and processed meat being related to increased and dairy and whole grains to reduced risk, while poultry, fish, eggs, nuts, refined grain products and soya are not clearly related to risk [6]. Given these inconsistencies, it remains unclear whether total protein intake resulting from these foods or the intake of protein from either animal-based or plant-based foods are related to T2D risk in humans.

Mechanisms that link the intake of dietary protein to the risk of T2D have to be related to one or both of the two known major pathophysiologic pathways of T2D: insulin resistance or impaired insulin secretion [8]. In particular, insulin resistance is closely associated with body fat accumulation, and effects of protein-rich diets may be indirectly linked to lower insulin resistance by their potential beneficial effects on body weight (BW). However, reductions in BW can be achieved by energy-restricted diets with different macronutrient composition [9, 10]. It is therefore important to clarify whether diets with different protein content affect insulin resistance, β-cell function and glycaemic status independent of energy intake and BW control (i.e. under isoenergetic settings). Dietary protein intake has been well established to augment postprandial insulin secretion, leading to enhanced glucose clearance from the blood by peripheral tissues [11]. However, such acute postprandial effects are not synonymous with long-term effects of protein-rich diets on tissue insulin sensitivity or secretory capacity of β-cells. Whether protein intake affects important glycaemic traits (glycated haemoglobin A1c [HbA1c], fasting glucose, fasting insulin) over longer periods of time (weeks to months) has been investigated in several RCTs. Overall, data from RCTs do not provide consistent evidence that high-protein intake [mostly around 30 energy percentage (%E)] or the choice of animal versus plant protein substantially affects major glycaemic traits [12–15].

Here, we present our findings from an umbrella review of systematic reviews (SRs) of prospective cohort studies and RCTs on protein intake (total as well as animal and plant protein) and the risk of T2D. We aimed to grade the overall certainty of evidence for such associations, considering the methodological quality of SRs, consistency of results, and biological plausibility. This work is part of a series of umbrella reviews on protein intake and health-related outcomes which are carried out as the basis of a new guideline on the effects of protein intake on health parameters by the German Nutrition Society [16].

## Methods

### Literature searches

Methodological details of umbrella reviews on protein intake and health-related outcomes which are carried out as the basis of a new guideline on the effects of protein intake on health parameters by the German Nutrition Society have been previously published (PROSPERO: CRD42018082395; [16]). The systematic literature searches were conducted in PubMed, Embase and Cochrane Database Systematic Reviews for SRs published between 01/07/2009 and 22/05/2022. The date of 07/2009 originates from the decision to cover a period of ten years, i.e. the initial database search was conducted in 11/07/2019, and the last update was made in 22/05/2022. The search strategy is presented in Supplementary Material S1. In addition to the systematic database search, the reference lists of the included SRs were reviewed for potentially relevant SRs. The literature searches were conducted independently by two authors. Any disagreements were resolved by consensus.

### Literature selection

Titles and/or abstracts of the retrieved studies were screened according to the pre-defined list of inclusion and exclusion criteria to identify potentially eligible SRs. The full manuscripts of these were retrieved and assessed for eligibility. It was tolerated that some of the primary studies were incorporated more than once in the included SRs. The literature selection process was conducted independently by two authors. Any disagreements were resolved by consensus.

The exclusion criteria are listed in detail in the description of our methodological approach [16] and in Supplementary Material S2. Briefly, SRs were included if they were related to general human populations (including seniors and amateur athletes), were published between 07/2009 and 05/2022, were SRs with or without meta-analysis (MA) of prospective studies (RCTs or prospective cohort studies, [including case-cohort or nested case–control studies]), were published in English or German language, considered high protein intake (if possible differentiated between proteins of plant and animal origin) as exposure and T2D as outcome. SRs were excluded if they considered studies with infants, children, adolescents, pregnant women, lactating women, or high-performance athletes, if the specific effect/association of protein was not assessed (e.g. whole food approach), if peptides (e.g. lactotripeptides) and/or single amino acids were investigated as exposures, and if relations between protein intake and T2D risk were not assessed. We also excluded articles on case studies, conference proceedings or articles which were only available in abstract form as well as umbrella reviews.

### Data extraction

Relevant data from each included SR were extracted into a standardised table. The data extraction was conducted independently by two authors. Any disagreement was resolved by discussion and consensus.

### Approaches to assess certainty and evidence

To reach a conclusion regarding protein intake and T2D, we proceeded in three steps. First, we assessed the methodological quality of retrieved SRs. Second, we used a scoring tool to assess the certainty of evidence of an association or effect between protein intake and T2D incidence. Third, we rated the overall certainty of evidence separately for each relevant exposure–outcome association considering all relevant SRs.

### Assessment of methodological quality and outcome-specific certainty of evidence

The methodological quality of the included SRs was assessed using a modified version of the “A Measurement Tool to Assess Systematic Reviews 2” tool (AMSTAR 2) [17]; Supplementary Material S3), which contains 14 items evaluating the methodological quality of the SR. The modifications are described in detail in our methodological protocol [16]. SRs were rated on a scale from high quality to critically low quality according to the existence of critical and non-critical methodological weaknesses. SRs graded as “critically low” by AMSTAR 2 were excluded from the rating of the overall certainty of evidence.

The outcome-specific certainty of evidence of included SRs was assessed using the NutriGrade scoring tool ([18]; Supplementary Material S4). NutriGrade aims to assess the certainty of evidence of an association or effect between different dietary factors and outcomes, taking into account nutrition research-specific requirements not considered by other tools. An important novelty of NutriGrade was the modified classification for MA of RCTs and cohort studies compared with the traditional GRADE approach (initially classifying RCTs with a high score and cohort studies with a low score) [16]. This tool utilises a numerical scoring system and comprises six items for SRs with MA of RCTs and eight items for MA of cohort studies. Based on a scoring system of a maximum of 10 points, the potential outcome-specific certainty of the evidence was rated based on four categories ranging from high (≥ 8 points) to moderate (6 to < 8 points) to low (4 to < 6 points) to very low (0 to < 4 points). Risk of bias contributes three points to the scale. The NutriGrade scoring tool was modified for the assessment of SRs without MA, as described in detail in Kroke et al. [16]. For SRs with or without MA reporting more than one relevant outcome, an assessment was conducted for each individual outcome. The methodological quality and outcome-specific certainty of evidence were assessed independently by two authors. Any disagreement was resolved by discussion and consensus.

### Grading the overall certainty of the evidence and deriving conclusions

The grading of the overall certainty of the evidence was assessed for each considered protein exposure-T2D association according to the criteria summarised in Supplementary Material S5. The overall grading ranges from convincing, probable, possible to insufficient. For this publication, two authors (MBS, SLO) made suggestions for rating the overall certainty of evidence. This rating was double-checked by a staff member of the German Nutrition Society (JH) and thereafter reviewed and approved by all co-authors. Grading of the overall certainty of evidence also requires a judgment about the biological plausibility of associations, To support such a decision, we considered SRs of RCTs related to glycaemic parameters (fasting blood glucose, fasting blood insulin, homeostatic model assessment insulin resistance [HOMA-IR], HbA_1c_) identified in the literature search. In case that such studies would not be consistent with a positive or negative association concluded from SRs on T2D risk, we concluded that the biological plausibility is not clearly given and therefore downgraded the overall certainty of evidence by one step. For example, if SRs on T2D risk supported a “probable” certainty of evidence for an increased risk of T2D with higher protein intake but SRs on glycaemic traits did not indicate a clear biological plausibility for this risk increase, we downgraded the overall certainty of evidence to “possible”.

## Results

**Fig. 1:**
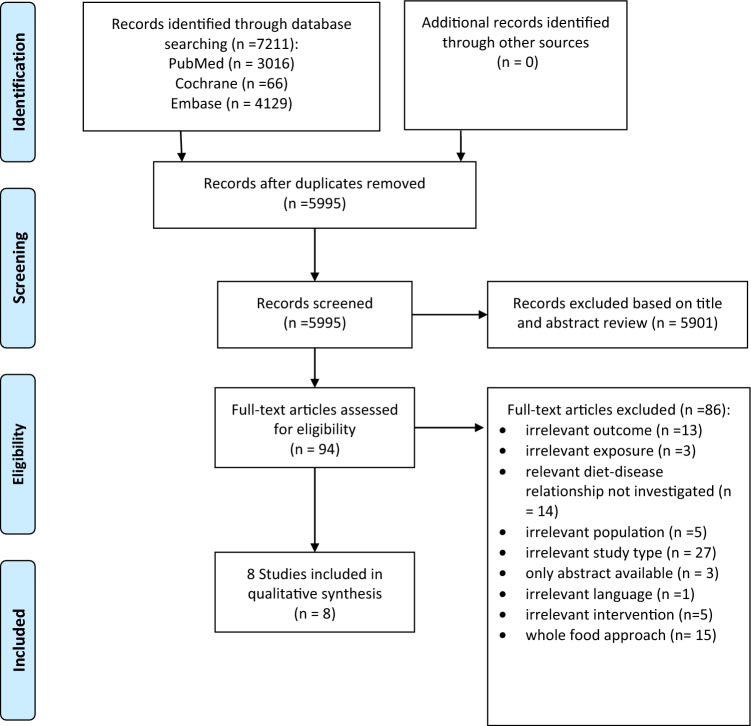
Flow diagram illustrating the identification and selection of systematic reviews

The literature selection process is outlined in Fig. 1. Of the 7211 publications initially identified, 94 were selected for full-text screening. A list of excluded SRs after full-text screening, including justifications for exclusion, is provided in Supplementary Material S6. One SR was excluded as it was written in a language that was not considered for our literature selection [19]. None of the SRs was excluded due to ‘critically low’ rating by AMSTAR 2. We finally selected eight SRs, which were published between 2012 and 2020 [13, 20–26].

### SR characteristics

Table 1 shows the characteristics of the included SRs. Whether modification of protein intake affects the risk of T2D has so far not been evaluated in SRs of RCTs, but rather in SRs of prospective observational studies, where habitual protein intake was investigated in relation to T2D risk: two SRs without MA [13, 24] and six SRs with MA [20–23, 25, 26], which included between three [26] and 12 prospective cohorts [25]. All SRs included evaluations of the intake of total protein, animal protein and/or plant protein in relation to the risk of T2D. The study follow-up period of the included primary studies ranged between 4.6 and 24 years. All SRs included participants from both sexes with an age between 20 to 80 years. The average intake amount and range of protein intake evaluated was not provided in most of the SRs. Three SRs reported average intakes of protein for comparison of extreme intake categories [13, 20, 24]. Based on the ten individual cohort studies of one SR, the protein intake was about 21% of total energy intake (*E*%) in the high intake group versus about 15 *E*% in the low intake group [20].

**Table 1:** Characteristics of SRs on type 2 diabetes

Author, year	Study type, study period	Study population	Exposition	Protein intake	Effect estimates: T2D incidence	Heterogeneity estimators	NutriGrade rating	AMSTAR 2 rating
Boushey, 2020 [24]	SR without MA of cohort studies published between 01/00 and 10/19, follow-up: 5–20 yrs	Age: NP, both sexes, healthy and/or at risk of chronic disease	Low vs. high protein intake	11.3–18.2 E% vs 13.1–21.6 E%	Inconsistent results:	NA		High
	12 studies	n = 448,344 participants	Total protein		∅: 6 estimates, ↑: 8 estimates, ↓: 1 estimate		Low (4)	
	10 studies	n = 396,137 participants	Animal protein		∅: 5 estimates, ↑: 5 estimates, ↓: 1 estimate		Low (4)	
	10 studies	n = 396,137 participants	Plant protein		∅: 10 estimates, ↑ 1 estimate		Moderate (5)	
Fan, 2019 [23]	SR with MA of prospective cohort studies published until 03/19, follow up: 7.2–20.1 yrs	Mean age: 21–75 yrs, both sexes	Dose–response (per 5E% increment in protein intake) and high *vs.* low protein intake	NP	RR (95% CI)			Moderate
	8 studies	n = 377,261 participants, cases = 20,211	Total protein		Per 5E%: 1.08 (1.05–1.11) High vs. Low: 1.11 (1.05 to 1.16)	*I*^2^: 0% *p* = 0.54 *I*^2^: 5% p = 0.40	Moderate (7.3)	
	11 studies	n = 350,536 participants, cases = 20,060	Animal protein		Per 5E%: 1.11 (1.07–1.15) High vs. Low: 1.13 (1.08–1.19)	*I*^2^: 43% *p* = 0.07 *I*^2^: 14% *p* = 0.31	Moderate (7.3)	
	11 studies	n = 317,848 participants, cases = 18,502	Plant protein		Per 5E%: 0.85 (0.76–0.96) High vs. Low: 0.93 (0.87 0.99)	*I*^2^: 42% *p* = 0.08 *I*^*2*^: 0% *p* = 0.48	Moderate (7.3)	
Ye, 2019 [25]	SR with MA of prospective cohort studies published until 11/18, follow up: 5–24 yrs	Age 20–79 yrs, both sexes	High vs*.* low protein intake	NP	RR (95% CI), random effect			High
	12 studies	n = 487,956 participants, cases = 38.350	Total protein		1.10 (1.03–1.17)	*I*^2^ = 49% *p* = 0.02	Moderate (6.8)	
	9 studies	n = 379,291 participants, cases = 33,011	Animal protein		1.13 (1.03–1.25)	*I*^2^ = 65% *p* = 0.002	Low (5.9)	
	9 studies	n = 379,291 participants, cases = 33,011	Plant protein		0.93 (0.86–1.01)	*I*^2^ = 32% *p* = 0.16	Low (5.9)	
Zhao, 2018 [22]	SR with MA of prospective cohort studies published until 03/18, follow up: 5–24 yrs (for one study not reported)	Age 20–80 yrs, both sexes, n = 440,418, cases = 34,221	High vs. low protein intake and dose–response analysis for a 5% of energy increment from protein	NP	RR (95% CI), random effect			High
	8 studies		Total protein—high vs. low		1.09 (1.02–1.15)	*I*^2^: 9% *p* = 0.37	Moderate (6.9)	
	9 studies		Total protein—per 5%E		1.09 (1.04–1.13)	*I*^2^: 42% *p* = 0.08		
	8 studies		Animal protein—high vs. low		1.19 (1.11–1.28)	*I*^2^ = 27% *p* = 0.21	Moderate (6.9)	
	8 studies		Animal protein—per 5%E		1.12 (1.08–1.17)	*I*^2^: 14% *p* = 0.32		
	8 studies		Plant protein—high vs. low		0.93 (0.87–0.99)	*I*^2^: 3% *p* = 0.41	Low (5.9)	
	8 studies		Plant protein—per 5%E		0.86 (0.75–1.00)	*I*^2^ = 34% *p* = 0.14		
Tian, 2017 [21]	SR with MA of prospective cohort studies published until 07/17, follow up: 5 to 24 yrs	Age 20–79 yrs, both sexes	High vs*.* low protein intake	NP	RR (95% CI), fixed effect			Low
	11 studies	n = 483,174 participants, cases = 52,637	Total protein		1.12 (1.08–1.17)	*I*^2^ = 19% *p* = 0.25	Low (5.3)	
	9 studies	n = 380,689 participants, cases = 31,557	Animal protein		1.14 (1.09–1.19)	*I*^2^ = 43% *p* = 0.30	Low (4.3)	
	9 studies	n = 381,879 participants, cases = 31,817	Plant protein		0.96 (0.88–1.06)	*I*^2^ = 59% *p* = 0.07	Low (4.4)	
Shang, 2016 [20]	SR with MA of prospective cohort studies published until 04/16, follow up: 5–24 yrs	Age: 20–79 yrs, both sexes	High vs*.* low protein intake		RR (95% CI), random effect	NA		Moderate
	10 studies	n = 468,315 participants, cases = 36,360	Total protein	14.37/15.15 E% vs. 20.85/21.67 E% (m/f)	1.09 (1.06–1.13)	*I*^2^ = 7% *p* = 0.38	Moderate (6.8)	
	8 studies	n = 395,959 participants, cases = 32,579	Animal protein	7.71/8.04 E% vs. 14.9/15.16 E% (m/f)	1.19 (1.11–1.28)	*I*^2^ = 26% *p* = 0.21	Low (5.4)	
	9 studies	n = 395,959 participants, cases = 32,579	Plant protein	4.43/4.85 E% vs. 8.19/8.66 E% (m/f)	0.95 (0.89–1.02)	*I*^2^ = 10% *p* = 0.35	Low (5.9)	
Pedersen, 2013 [13]	SR without MA of RCTs and prospective cohort studies published 01/00–12/11, follow up: 9–20 yrs	4 studies n = 188,695 participants	High *vs.* low protein intake	14.7/15.7 E% vs. 18.4/21.5 E% (2 studies)	“The evidence is assessed as suggestive regarding the relation of total and animal protein intake to increased risk of T2D, based on long-term LCHP diets, including one study with an LCHP-high-fat diet, while evidence is assessed as inconclusive regarding the relation of total protein to fasting glucose (Table 9)”	NA		Moderate
			Total protein				Moderate (5.0)	
			Animal protein				Low (3.0)	
			Plant protein				Low (4.0)	
Alhazmi, 2012 [26]	SR with MA of prospective cohort studies published until 07/12, follow up: 6 to 20 yrs	Age: 21–78 yrs, both sexes	High vs*.* low protein intake		RR (95% CI), random effects			Low
	3 studies	n = 207,513 participants, cases = 6,290	Total protein		1.02 (0.91–1.15)	*I*^2^ = 0% *p* = 0.45	Low (4.5)	
	3 studies	n = 160,462 participants, cases = 7,146	Animal protein		1.17 (0.94–1.47)	*I*^2^ = 72% *p* = 0.03	Low (4.5)	
	3 studies	n = 160,462 participants, cases = 7,146	Plant protein		0.99 (0.87–1.12)	I^2^ = 0% p = 0.41	Low (4.5)	

*AMSTAR 2* A measurement tool to assess systematic reviews 2, *CI* confidence interval, *d* day(s), *E%* energy percentage, *f* female, *HR* hazard ratio, *m* male, *MA* meta-analysis, *NA* not applicable, *NP* not provided, *RR* relative risk, *SR* systematic review, *wk* week, *yr* year

An overview of all primary studies included in the eight SRs [20, 27–41] is provided in Supplementary Material S7. Noteworthy, the MA by Alhazmi et al. [26] included not only the lowest number of individual studies (three), this MA included two analyses from the same cohort study, i.e. the Nurses’ Health Study [27, 29]. Furthermore, the MA by Tian et al. [21] included two analyses of the Nurses’ Health Study [34, 40], with one publication investigating protein intake in relation to gestational diabetes rather than T2D [34]. For the remaining four SRs with MA, there is high overlap with respect to the individual cohort studies included (Supplementary Material S7). All four SRs included analyses of the Melbourne Collaborative Cohort [20] as well as the Nurses’ Health Study, the Nurses’ Health Study II and the Health Professionals Follow-up Study [40]. Three out of the four MA considered also the Women’s Health Initiative [33], the Australian Longitudinal Study on Women’s Health [36], the European Prospective Investigation into Cancer and Nutrition [37], and the Japan Public Health Center-Based Prospective Study [38]. SRs differed with respect to the consideration of the EPIC-NL Study cohort [32] and the Malmö Diet and Cancer cohort [35], which were not considered by Zhao et al. [22]. Both cohorts are part of the EPIC cohort, and thus their data contributed to the analysis of van Nielen et al. [37]. The MA by Shang [20] and Ye et al. [25] therefore included these populations twice. Also, data from the Kuopio Ischaemic Heart Disease Risk Factor Study [39] were published after the MA by Shang et al. [20], and were thus included only in later MA [22, 23, 25]. The most recent MA by Fan et al. [23] included a more recent cohort study [41], which is not included in previous MA, but did not include several other cohort studies which were included in previous MA (e.g. EPIC, Women’s Health Initiative [33]). While the SR without MA by Pedersen et al. [13] included only four cohort studies, all published until 2010, the recent SR without MA by Boushey et al. [24] included 12 primary studies.

### Methodological quality

Overall scores of AMSTAR 2 for each SR included are summarised in Table 1; Supplementary Material S8 provides more detailed information of this quality assessment. Methodological quality was rated low for two SRs [21, 26] but the majority of SRs was rated moderate [13, 20, 23] or high [22, 24, 25].

### Evaluation of biological plausibility

To address the biological plausibility for a positive association between higher total protein intake and T2D risk, we evaluated several MA which summarised results from dietary RCTs on major glycaemic traits (Supplementary Material S9) [12, 15, 42–48]. The MA by Santesso et al. [15] included RCTs which compared dietary interventions with a difference of protein intake by at least 5% total energy intake over a duration of 1–12 months. High-protein diets provided on average (median) 27 E% from protein, while lower protein diets contained 18 E%. The MA showed no difference between higher and lower protein diets on HbA_1c_ (three RCTs, mean difference: 0.00 units) and fasting glucose (15 RCTs, standardised mean difference: − 0.05 units). However, higher protein diets significantly reduced fasting insulin concentrations by on average 0.20 units compared to lower protein diets (11 RCTs). Similarly, the MA by Schwingshackl [12], which included RCTs with durations between 12 and 24 months, revealed lower fasting insulin levels after high-protein diets (≥ 25 E%) compared to lower protein diets (≤ 20 E%) [11 RCTs, mean difference between diets: − 0.71 µIU/ml (95% CI − 1.36 to − 0.05)]. Effects on fasting glucose (11 RCTs, mean difference: − 0.63 mg/dl between the protein intervention arms) and HbA_1c_ (3 RCTs, mean difference: 0.07%) were not statistically significant. A SR without MA concluded that there is insufficient evidence for a link between total protein intake and fasting glucose [13]. While there is evidence from RCTs that high-protein diets reduce fasting insulin concentrations more than lower protein diets do, this reduction in insulin resistance does neither translate into differences in glycaemic status as measured by fasting glucose and HbA_1c_ nor is it consistent with an increased risk of T2D.

Based on these data, we conclude that there is no biological plausibility for the association between T2D and protein intake. Since this is a key component of the overall certainty of evidence assessment, we downgrade the derived strength of evidence by one level each.

### Overall certainty of the evidence for associations of protein intake with risk of T2D

#### Total protein

The vast majority of SRs with MA (five out of six) reported an increased risk of T2D with higher total protein intake [20–23, 25]. The only MA not reporting an association [26] included considerably fewer individual studies (three) than the other MA (more than eight) and, thus, had lower precision, and was rated with a low certainty of evidence by NutriGrade (Table 1, Supplementary Material S10). While the association between protein intake and T2D risk in the SR by Tian et al. [21] was also rated as “low” certainty, in the remaining four SRs with MA the certainty was rated as “moderate” and the methodological quality (with AMSTAR 2) as “moderate” or better [20, 22, 23, 25]. Shang et al. evaluated results from 10 prospective cohort studies [20]. The pooled relative risk (RR) for T2D for the comparison of the highest with the lowest categories of total protein intake was 1.09 (95% CI 1.06–1.13), and there was low statistical heterogeneity (*I*^2^ = 7%). Quite similar positive associations have been reported in the most recent three MA. Zhao et al. (2018) [22] reported a RR of 1.09 (95% CI 1.04–1.13) for a 5% energy increment from protein and a RR of 1.09 (95% CI 1.02–1.15, *n* = 8 cohort studies) comparing high versus low intake. Ye et al. [25] reported a RR of 1.10 (95% CI 1.03–1.17, *n* = 12 cohorts) comparing high versus low intake categories. Most recently, Fan et al. 2019 [23] reported a RR of 1.08 (95% CI 1.05–1.11) for a 5% energy increment from protein and a RR of 1.11 (95% CI 1.05–1.16) comparing high versus low intake categories based on nine cohort studies (12 estimates considering subgroups). While two MA observed a moderate degree of statistical heterogeneity (*I*^2^ = 42% in Zhao et al. [22], 49% in Ye et al. [25]), heterogeneity across the studies was statistically significant only in one of them (*p* = 0.020) [25]. In this case statistical heterogeneity may be explained by geographical location (i.e. the lack of associations in Asian cohorts) as well as study quality (lack of associations in studies with lower quality) [25]. Noteworthy, despite this indication of heterogeneity, the vast majority of primary studies included in these SRs points towards a positive association [22, 25]. In contrast, statistical heterogeneity was lacking in the MA by Fan et al. (*I*^2^ = 0%) [23]. The SR by Pedersen et al. [13], which was based on four prospective cohort studies, concluded that there is suggestive evidence that total protein intake increases T2D risk, but the SR did not summarise individual study findings by MA. The SR without MA by Boushey et al. [24] included twelve individual cohort studies and the association between protein intake and T2D risk was rated as “low” with NutriGrade. Eight statistically significant positive associations, one significant inverse association and six non-significant associations were found, partly considering estimates for subgroups within individual cohorts. Boushey et al. [24] concluded that there is insufficient evidence for associations between total protein intake and T2D risk.

In summary, the majority of MA of cohort studies (five out of six) [20–23, 25, 26], particularly those with large numbers of individual studies (five out of five) [20–23, 25], observed consistently a higher T2D risk with higher total protein intake. In the majority of MA (four out of six) the outcome-specific certainty of evidence was rated as “moderate” by NutriGrade, and the methodological quality at least as “moderate” [20, 22, 23, 25]. Two SRs without MA concluded that there is either suggestive or insufficient evidence for an association of total protein intake with risk of T2D [13, 24].

Thus, the methodological quality of the identified SRs and the outcome-specific certainty of evidence from these SRs would results in an overall certainty of evidence rated as “probable”. There is, however, no strong support for the biological plausibility for the observed associations. We therefore concluded that possible evidence exists that higher protein intake increases the risk of T2D (Table 2).

**Table 2:** The overall certainty of evidence: Effect of dietary protein intake on T2D incidence

	Reference	Study type	Total protein and T2D incidence [n = number of studies]	AMSTAR 2 rating^1^	NutriGrade rating^2^
Total Protein	Boushey 2020 [24]	SR	n = 12 Ø	High	Low
	Fan 2019 [23]	MA	n = 8 ↑	Moderate	Moderate
	Ye 2019 [25]	MA	n = 12 ↑	High	Moderate
	Zhao 2018 [22]	MA	n = 8 ↑	High	Moderate
	Tian 2017 [21]	MA	n = 11 ↑	Low	Low
	Shang 2016 [20]	MA	n = 10 ↑	Moderate	Moderate
	Pedersen 2013 [13]	SR	n = 4 ↑	Moderate	Moderate
	Alhazmi 2012 [26]	MA	n = 3 Ø	Low	Low
	∑	n = 8 ↑ (n = 6); Ø (n = 2) Probable link for an increase in risk between total protein intake and T2D incidence			
		Justified by the lack of biological plausibility, the degree of hardness is downgraded by one level and thus lies at possible			

	Reference	Study type	Animal protein and T2D incidence [n = number of studies]	AMSTAR 2 rating^1^	NutriGrade rating^2^
Animal Protein	Boushey 2020 [24]	SR	n = 10 Ø	High	Low
	Fan 2019 [23]	MA	n = 11 ↑	Moderate	Moderate
	Ye 2019 [25]	MA	n = 9 ↑	High	Low
	Zhao 2018 [22]	MA	n = 8 ↑	High	Moderate
	Tian 2017 [21]	MA	n = 9 ↑	Low	Low
	Shang 2016 [20]	MA	n = 8 ↑	Moderate	Low
	Pedersen 2013 [13]	SR	n = 4 ↑	Moderate	Low
	Alhazmi 2012 [26]	MA	n = 3 Ø	Low	Low
	∑	n = 8 ↑ (n = 6); Ø (n = 2) Possible link for an increase in risk between animal protein intake and T2D incidence			
		Justified by the lack of biological plausibility, the degree of hardness is downgraded by one level and thus lies at insufficient			

	Reference	Study type	Plant protein and T2D incidence [n = number of studies]	AMSTAR 2 rating^1^	NutriGrade rating^2^
Plant Protein	Boushey 2020 [24]	SR	n = 10 Ø	High	Moderate
	Fan 2019 [23]	MA	n = 11 ↓	Moderate	Moderate
	Ye 2019 [25]	MA	n = 9 Ø	High	Low
	Zhao 2018 [22]	MA	n = 8 ↓	High	Low
	Tian 2017 [21]	MA	n = 9 Ø	Low	Low
	Shang 2016 [20]	MA	n = 9 Ø	Moderate	Low
	Pedersen 2013 [13]	SR	n = 4 Ø	Moderate	Low
	Alhazmi 2012 [26]	MA	n = 3 Ø	Low	Low
	∑	n = 8 Ø (n = 6); ↓ (n = 2) Possibly no link between plant protein intake and T2D incidence; Insufficient evidence for an inverse association of plant protein intake and risk of T2D			

*AMSTAR 2* A measurement tool to assess systematic reviews 2, *MA* systematic review with meta-analysis, *SR* systematic review without meta-analysis, *T2D* type 2 diabetes mellitus, *Ø* inconsistent results, ↑ increased risk, ↓ decreased risk

^1^Shea et al. [17]; Supplement S8

^2^Schwingshackl et al. [18]; Supplement S10

#### Animal protein

Higher animal protein intake has been evaluated with regard to the risk of T2D in six SRs with MA of prospective cohort studies [20–23, 25, 26] (Table 1). Estimates point toward a higher T2D risk with high protein intake in the analysis by Alzhami et al. [26], but it included only three cohort estimates resulting in a lack of precision (1.17; 95% CI 0.94–1.47). This MA was considered as of low quality and two of the included cohorts were from the same study population. Positive statistically significant associations were observed in the remaining five MAs [20–23, 25]. Shang et al. [20] reported a RR of 1.19 (95% CI 1.11–1.28, *n* = 8 cohort studies) comparing highest with lowest animal protein intake categories. Tian et al. [21] included nine cohort studies with an association of 1.14 (95% CI 1.09–1.19) comparing highest with lowest categories of animal protein. Ye et al. [25] summarised RRs for the comparison of highest versus lowest categories of animal protein intake, with a pooled RR of 1.13 (95% CI 1.03–1.25). Statistical heterogeneity exists between studies (*I*^2^ = 65%), with geographical region and study quality being main determinants of this heterogeneity, similar to the investigation of total protein intake [25]. However, the association in these three MA [20, 21, 25] were rated as low certainty (Table 1, Supplementary Material S10). The remaining two MA by Zhao et al. [22] and Fan et al. [23] were graded at least “moderate” by both NutriGrade and AMSTAR 2 and included each eight cohort studies. Zhao et al. [22] reported a point estimate of 1.12 (95% CI 1.08–1.17) for a 5% of energy increment from animal protein (1.19; 95% CI 1.11–1.28 for comparison of high versus low intake categories). Fan et al. [23] reported a RR of 1.11 (95% CI 1.07–1.15) for a 5% of energy increment from animal protein. In both SRs, there was no significant statistical heterogeneity and point estimates of primary studies included all pointed towards a positive association. The SR by Boushey et al. [24] included 9 individual cohort studies and concluded that there is insufficient evidence for a relation of animal protein intake with risk of T2D, but did not summarise study results by MA.

The majority of MA of cohort studies (five out of six) [20–23, 25, 26], particularly those with large numbers of individual studies (five out of five) [20–23, 25], observed a consistently higher T2D risk with higher animal protein intake. However, most MA (four out of six) were rated with a “low” certainty by NutriGrade [20, 21, 25, 26] and only two were rated with a “moderate” certainty of evidence [22, 23]. Similar ratings were detected for the methodological quality [22, 23]. Six out of eight SRs were graded “moderate” or “high” by AMSTAR 2 [13, 20, 22–25]. One SR without MA, rated “low” with NutriGrade [24], concluded that there is insufficient evidence for a relation. Thus, the low methodological quality of the majority of SRs and the slightly inconsistent certainty of evidence from these SRs would result in an overall certainty of evidence rated as “possible”.

However, there is no clear biological plausibility for the observed associations. RCTs comparing animal and plant protein did not reveal clear differences: according to an SR without MA [14] (Supplementary Material S9), most intervention studies did not observe differences in the effects of animal versus plant protein on fasting insulin, fasting glucose or HOMA-IR. There was no indication that effects would be markedly different if protein intake from different animal and plant food sources are compared. As pointed out above, there is also no convincing support that animal protein intake would indirectly—by an effect via higher total protein intake—affect major glycaemic parameters related to T2D in a way being consistent with an increased risk with higher intake. We therefore conclude that, in the absence of clear biological plausibility, there is insufficient evidence for a positive association (Table 2).

#### Plant protein

Several SRs of prospective cohort studies with MA have evaluated whether the intake of plant protein is associated with the risk of T2D. While Alzhami et al. [26] and Tian et al. [21] did not observe an association between plant protein intake and the risk of T2D; these associations were rated with a “low” certainty by NutriGrade, and low methodological quality by AMSTAR 2 (Table 1). Higher plant protein intake was slightly inversely related to T2D risk in the MA by Shang et al. [20] and Ye et al. [25]; however, 95% CIs overlapped 1.00 (RR 0.95; 95% CI 0.89–1.02 and 0.93; 95% CI 0.86–1.01, respectively, comparing high versus low categories of intake). However, Zhao et al. [22] reported a RR of 0.93 (95% CI 0.87–0.99) for a similar comparison in their MA. Modelling plant protein intake as a 5% increment of energy intake resulted in a RR of 0.86 (95% CI 0.75–1.00), without evidence for statistical heterogeneity. Similarly, Fan et al. [23] reported an inverse association of plant protein intake, with a RR of 0.85 (95% CI 0.76–0.96, *n* = 11 cohort studies) for each 5% increment of energy intake. Three MA reported non-linear associations for plant protein intake, with the lowest risk observed for moderate intake levels: In the MA by Ye et al. [25] moderate intake (intake categories other than the lowest and highest intake categories in each study) was related to statistical significantly lower risk of T2D (RR: 0.94; 95% CI 0.92–0.97) compared to low intake. Similarly, the MA by Zhao et al. [22] and Fan et al. [23] observed a U-shaped relationship, with the maximum reduction observable at about 5–6% of energy from plant protein intake.

All MA received a “low” NutriGrade rating [20–22, 25, 26], with the exception of Fan et al. [23], which was rated as “moderate”. The SR without MA by Boushey et al. [24], rated as “moderate” by NutriGrade and “high” by AMSTAR 2, included nine individual cohort studies [20, 30, 32, 35, 37–41] and concluded that there is insufficient evidence for a relation of plant protein intake with risk of T2D. Although some MA point towards a reduced risk of T2D at higher plant protein intakes, with the suggestion of non-linear associations, the majority of SRs does not support a clear relation. Also, in the vast majority of SRs the association between plant protein and T2D risk was rated with a “low” certainty by NutriGrade [13, 20–22, 25, 26].

Thus, possible evidence exists for the lack of a relationship between plant protein intake and T2D incidence (Table 2). An inverse association, suggested by some more recent SRs, lacks consistency. Also, given that plant protein (as compared to animal protein) seems not to affect major glycaemic traits in RCTs [14] (Supplementary Material S9), there is no strong support that an association with T2D risk is plausible. These points, together with the mostly low outcome-specific certainty of evidence of SRs, led us to conclude that there is insufficient evidence that higher plant protein intake lowers the risk of T2D (Table 2).

## Discussion

In the present umbrella review, we identified eight SRs that evaluated an association between protein intake (total, animal and plant protein, respectively) and T2D risk, of which six also provided estimates from MAs. Overall, positive associations for total and animal protein and the risk of T2D were observed in the majority of SRs. However, the results of RCTs on important glycaemic traits do not provide strong support that total and animal protein intake adversely impacts the pathogenesis of T2D. In light of the lack of biological plausibility and the low outcome-specific certainty by NutriGrade for SRs, in particular for animal protein, the overall certainty of evidence for an increased T2D risk was judged to be possible for high total protein and insufficient for high animal protein intake. While some SRs support a lower T2D risk with higher plant protein intake, SRs findings were mainly inconsistent. This, together with the lack of a clear biological plausibility as indicated by a lack of effect on major glycaemic traits in RCTs, led us to conclude that there is insufficient evidence for such an inverse association. Given that the majority of SRs do not point out an association, the overall evidence that there is no association was considered possible.

Whether protein intake is associated with T2D risk has been evaluated in three umbrella reviews of SRs. Bellou et al. [49] reviewed SRs that evaluated the association of various dietary and non-dietary factors with T2D, but identified only the SR by Alhazmi et al. [26], which analysed the relation between protein intake and T2D risk. As discussed above, this SR included notably fewer individual studies than more recent SRs and its quality was rated low. Neuenschwander et al. [6] conducted an umbrella review of dietary risk factors for T2D and concluded that there is evidence that total and animal protein intakes are related to increased T2D risk. The certainty of evidence was graded as moderate for both total and animal protein in this umbrella review, while we graded the evidence to be possible for total protein intake but insufficient for animal protein intake. Similar to our umbrella review, individual SRs were graded with AMSTAR and the certainty of evidence was evaluated with a modified version of NutriGrade. However, in contrast to our review, this umbrella review included a re-analysis (random effects MA) of the original studies identified in the SRs. Based on nine primary cohort studies, each 5% increase in energy from total protein intake was related to an RR of 1.09 (95% CI 1.04–1.13). The respective estimate for animal protein, based on eight primary studies, was 1.12 (95% CI 1.08–1.17). For plant protein no statistically significant association with T2D risk was observed (RR per 5%*E* 0.87; 95% CI 0.74–1.01), and the certainty of evidence was rated as “low” [6]. In contrast to our umbrella review, only one SR was selected for further evaluation for each dietary exposure, e.g. based on the largest number of individual studies or of T2D cases included. Thus, while the SRs by Shang et al. [20] and Tian et al. [21] were also identified in this umbrella review [6], only the SR by Zhao et al. [22] was selected. More recent SRs by Ye et al. [25] and Fan et al. [23] were not included in the umbrella review by Neuschwander et al. [6], likely because of the later time of publication. Lv et al. have reviewed SRs on protein intake and multiple health outcomes, including T2D [50]. The authors included 2 SRs (Zhao et al. [22], Ye et al. [25]). While more SR were identified [13, 20, 23, 26], the authors included only those SRs which reported the highest number of primary studies. The two included SRs were graded to have “high” methodological quality using AMSTAR, similar to our AMSTAR 2 grading. Lv et al. reported “highly suggestive evidence” for a relationship of higher animal protein intake and higher T2D risk for the SR by Zhao et al. [22], while the evidence was classified as “weak” for the SR by Ye et al. [25]. Suggestive evidence was found for a positive relationship of total protein and T2D risk and for an inverse association of moderate plant protein intake. But again, this evidence classification refers to a single SR each, and the corresponding second SR was classified as either “weak” or “not significant”. The evidence classification by Lv et al. considered statistical significance and precision, number of cases, heterogeneity, evidence for small study effects, and evidence for excess significance bias. Lv et al. [50] did not reveal the evidence from individual SRs to an overall certainty of evidence as we did in our UR. Our umbrella review and certainty of evidence evaluation therefore included considerably more SRs than previous umbrella reviews on the topic.

While SRs on total protein intake quite consistently observed higher T2D risk at higher protein intake, this association seems to be restricted to animal protein only, given that animal protein but not plant protein was found to be positively associated with T2D risk. Such a difference could be related to the relative abundance of different amino acids in animal versus plant protein. Animal protein provides higher amounts of branched-chain amino acids compared to plant proteins, and circulating leucine, isoleucine, and valine may be risk factors for T2D [51]. Furthermore, circulating glycine was found to be associated with a higher risk of T2D; as it is abundant in animal protein, it has been considered as a potential mediator which links higher intake of red meat to T2D [52]. In addition, higher circulating tyrosine levels appear to be causally related to reduced T2D risk [53]; nevertheless, tyrosine is an abundant amino acid in both animal and plant foods and may unlikely explain the contrasting associations found for animal versus plant protein intake in cohort studies. However, despite some evidence that the amino acid composition of animal protein may be relevant for the pathogenesis of T2D, we downgraded the overall certainty of evidence for all protein exposures due to the lack of evidence that changes in total protein intake or the relative proportion of animal versus plant protein showed mid- to longer-term effects on major glycaemic traits in RCTs. The reasons for the discrepancy between RCTs on glycaemic traits and long-term cohort studies on T2D incidence remain unclear. Generally, protein intake in observational studies is estimated from food intake and associations observed may not necessarily reflect causal effects of the nutrient per se. It is rather possible that protein intake is a marker for specific protein-rich foods. In this context, associations of protein-rich animal foods with T2D risk are not homogenous [2, 3, 6]; positive associations have largely been restricted to unprocessed red meat and processed meat. This makes it questionable that animal protein intake per se could have detrimental effects. Further, associations found for red meat may be explained by other food constituents than protein [52]. Finally, residual confounding cannot be excluded as alternative explanation for observational study results.

There are several other limitations inherent to observational studies on dietary risk factors for T2D. Observational studies rely on self-reported dietary intake which is generally prone to misreporting. Cohort studies frequently rely on semi-quantitative assessment instruments like food frequency questionnaires, which are prone to measurement error and not designed to provide an accurate quantitative estimate of absolute protein intake. For example, correlations between protein intake estimated by questionnaires and urinary nitrogen excretion (the gold standard for validating self-report instruments) range from 0.07 to 0.57 [54]. Given that measurement errors are unrelated to the disease status during follow-up in cohort studies, they would likely tend to lead to an underestimation of the true association. This is indicated by results of the Women’s Health Initiative which is included in several of the identified SRs: the association of total protein intake (as *E*%) with the risk of T2D was markedly stronger when corrected for this measurement error using regression calibration [33]. Furthermore, investigations on macronutrient composition generally reflect substitution effects under isoenergetic settings, but SRs provide usually only limited information whether associations included in MA refer to substitutions of protein for total CHO and/or total fat, if subgroups of CHO or fat were considered (e.g. low versus high quality CHO, saturated vs. unsaturated fatty acids), or if higher animal or plant protein intake reflect also a higher total protein intake or rather a substitution of one for the other—although the relevance of model choice to reflect specific macronutrient substitutions in relation to T2D risk has been well documented [30, 55].

However, the limitation of current RCTs in relation to glycaemic traits might be also important. While an effect of protein intake on fasting insulin was shown, it is noteworthy that this may not reflect the effect to be expected in isoenegetic settings. The interventions of most RCTs included in the MA by Santesso et al. [15] and Schwingshackl et al. [12] involved energy-restricted weight-loss diets and several studies found different effects on BW. Thus, it remains unclear from the MA to which extent reductions in fasting insulin can be explained by the differences in weight-loss between the study arms. It is also noteworthy that high-protein interventions were applied in many of the included RCTs (often around 30 *E*% from protein), while there are apparently no MA of RCTs that investigate protein intake at the higher end of habitual intake (between 18 and 25 *E*%). Thus, the generalizability of findings on protein intake and biomarkers of glucose homeostasis from RCTs to real-world settings is limited. The PREVIEW RCT, which compared a high protein (25%*E*) and low glycaemic index diet with a moderate protein (15%*E*) and moderate glycaemic index diet among persons with prediabetes in a three-year weight maintenance intervention following an eight-week weight reduction, did not observe an effect on T2D incidence [56]. However, incidence was overall low in the study, limiting the power for group comparisons. Interestingly, significantly fewer participants achieved normoglycaemia at three years in the high protein compared to the moderate protein group, although weight loss was comparable [56].

The comprehensive and systematic literature search as well as the assessment of the methodological quality of the SRs with AMSTAR 2 and the rating of the outcome-specific certainty of evidence with NutriGrade are clear strengths of the current umbrella review. Next, all methodological steps of the review procedure have been defined a priori as described [16]. Furthermore, we included evidence of all identified SRs in our overall certainty of evidence assessment. However, our procedure requires relatively high certainty of evidence in all or most individual SRs to result in high overall certainty of evidence. Our literature search revealed several SRs on the topic, but with different coverage of individual studies and with varying quality assessment. We applied NutriGrade instead of the GRADE approach (Grading of Recommendations, Assessment, Development and Evaluation) because an important novelty of NutriGrade (published in 2016) was the modified classification for MA of RCTs and cohort studies compared with the traditional GRADE approach (initially classifying RCTs with an initial high score and cohort studies with a low score) [57]. We are aware that in the meantime the GRADE approach was amended (adjustments published in 2019, but after the guideline methodology was established in 2017) in a way that cohort studies can now also be assigned an initially high score, when risk of bias tools such as ROBINS-I are used [58]. Furthermore, some reviews included individual cohort populations twice or included publications on endpoints other than T2D. Restricting the certainty of evidence assessment to the most recent or comprehensive SRs or to those which meet a pre-defined quality threshold may lead to a higher evidence grade. For example, the SR by Zhao et al. covers all individual cohorts identified also by any other SR except one, not considering duplicate publications from the same cohort population. This SR was rated “high” by AMSTAR 2 and “moderate” by NutriGrade. We also did not consider a re-analysis of original studies, although it is clear that none of the SRs included all relevant individual studies.

An umbrella review also has limitations. For example, more recent primary studies cannot be included in the evaluation and are therefore not taken into account. In addition, there is a dependency on the inclusion and exclusion criteria of the underlying SRs; for the umbrella review, the largest possible overlap with its own inclusion and exclusion criteria must be achieved. Accordingly, important results may not be taken into account because the inclusion criteria are not completely fulfilled. The quality of the umbrella review is largely dependent on the quality of the SRs and thus on the quality of the primary studies. Under certain circumstances, the primary studies included in the reviews differ considerably from one another, so that the informative value of the umbrella review is limited. In addition, it is dependent on the summary of findings at the level of the SRs, whereby outcome, subject structures and/or the interventions may be standardised or summarised. On the other hand, umbrella reviews have strengths. Umbrella reviews summarise the best possible evidence so that SRs with and without MA can be summarised. Umbrella reviews are considered as the highest level of evidence. The amount of available evidence is becoming more and more heterogenous, so umbrella reviews are a good way to summarise the available data [59, 60].

## Conclusions

The present umbrella review reveals that there is possible overall certainty that a high total protein intake increases the risk of T2D. The SRs with and without MA included in this umbrella review found quite consistently such associations between total protein and T2D risk. However, this evidence from observational cohort studies is not supported by results from human intervention studies evaluating effects of protein intake on major glycaemic traits. While a higher T2D risk was also reported quite consistently for high animal protein intake, most SRs had only low certainty of evidence for such an association. Also considering the lack of a clear biological link, the overall certainty of evidence for a risk increase with high animal protein intake is insufficient. While some SRs support a lower T2D risk with higher plant protein intake, the lack of consistent evidence, the low methodological quality of most SRs and the lack of a clear biological plausibility reflects insufficient overall certainty of evidence for such a risk reduction. Given that the majority of SR did not indicate an association, there is possible overall certainty of evidence for a lack of association.

### Supplementary Information

Below is the link to the electronic supplementary material.

Supplementary file1 (DOCX 18 KB)

Supplementary file2 (DOCX 20 KB)

Supplementary file3 (DOCX 32 KB)

Supplementary file4 (DOCX 61 KB)

Supplementary file5 (DOCX 16 KB)

Supplementary file6 (DOCX 30 KB)

Supplementary file7 (DOCX 18 KB)

Supplementary file8 (DOCX 18 KB)

Supplementary file9 (DOCX 23 KB)

Supplementary file10 (XLSX 31 KB)

Supplementary file11 (XLSX 34 KB)

## Abbreviations

AMSTAR 2: A measurement tool to assess systematic reviews 2﻿
BW: Body weight
CHO: Carbohydrate
%E: Energy percentage
HbA1c: Glycated haemoglobin A1c
﻿HOMA-IR: Homeostatic model assessment insulin resistance
MA: Meta-analysis/meta-analyses
RCT: Randomised controlled trial
RR: Relative risk
SR: Systematic review
T2D: Type 2 diabetes mellitus
