# Supplementary material for: Protein intake and type 2 diabetes mellitus: an umbrella review of systematic reviews for the evidence-based guideline for protein intake of the German Nutrition Society
Source: Eur J Nutr. 2023 Sep 17;63(1):33–50. doi: 10.1007/s00394-023-03234-5 (PMC10799123; doi:10.1007/s00394-023-03234-5)
Supplement: Supplementary file 1 — Supplementary file1 (DOCX 18 KB) [file 394_2023_3234_MOESM1_ESM.docx]

Supplementary Material S1 Search strategy for systematic literature search.

|  | **Database** | | |
| --- | --- | --- | --- |
| **Research**  **topic** | **PubMed** | **Cochrane Database of Systematic Reviews** | **Embase** |
| **Study types** | Meta-analy* [tiab] OR "meta-analysis" [tiab] OR "meta analyses" [tiab] OR "meta analysis" [tiab] OR metaanalysis [tiab] OR "meta-analyze" [tiab] OR "meta-analysis" [Publication Type] OR systematic [sb]^1^ OR "systematic review" [tiab] | - | 'Meta analysis'/exp OR 'systematic review'/exp OR meta-analy*:ti,ab OR 'meta-analysis':ti,ab OR 'meta analyses':ti,ab OR 'meta analysis':ti,ab OR metaanalysis:ti,ab OR 'meta-analyze':ti,ab OR 'systematic review':ti,ab |
| **Protein** | "dietary proteins" [mh] OR "diet, protein-restricted" [mh] OR "whey proteins" [mh] OR protein [tiab] OR proteins [tiab] OR "high-protein" [tiab] OR "low-protein" [tiab] OR "whey powder" [tiab] OR "whey powders" [tiab] OR "hypoprotein diet" [tiab] OR "peptidyl group" [tiab] OR "dairy product" [tiab] OR "dairy products" [tiab] OR "protein-free" [tiab] OR "protein-restricted" [tiab] | [mh "dietary proteins"] OR [mh "diet, protein-restricted"] OR [mh "whey proteins"] OR protein:ti,ab OR proteins:ti,ab OR "high-protein":ti,ab OR "low-protein":ti,ab OR "whey powder":ti,ab OR "whey powders":ti,ab OR "hypoprotein diet":ti,ab OR "peptidyl group":ti,ab OR "dairy product":ti,ab OR "dairy products":ti,ab OR "protein-free":ti,ab OR "protein-restricted":ti,ab | 'protein intake'/exp OR 'protein restriction'/exp OR 'dairy product'/exp OR 'yolk protein'/exp OR 'proteins by anatomical concept'/exp OR 'proteins by organism'/exp OR protein:ti,ab OR proteins:ti,ab OR 'high-protein':ti,ab OR 'low-protein':ti,ab OR 'whey powder':ti,ab OR 'whey powders':ti,ab OR 'hypoprotein diet':ti,ab OR 'peptidyl group':ti,ab OR 'dairy product':ti,ab OR 'dairy products':ti,ab OR 'protein-free':ti,ab OR 'protein-restricted':ti,ab |
| **Diabetes** | "Diabetes mellitus, type 2" [mh] OR "glycated hemoglobin A" [mh] OR Glucose [mh] OR "Insulin Resistance" [mh] OR diabet* [tiab] OR "glycated haemoglobin A" [tiab] OR "glycated hemoglobin A" [tiab] OR "glycosylated haemoglobin A" [tiab] OR "glycosylated hemoglobin A" [tiab] OR glucose [tiab] OR glycemic [tiab] OR glycaemic [tiab] OR "insulin sensitivity" [tiab] OR "insulin resistance" [tiab] OR prediabetes [tiab] OR pre-diabetes [tiab] OR prediabetic [tiab] OR pre-diabetic [tiab] OR NIDDM [tiab] OR T2DM [tiab] OR T2D [tiab] OR HbA1 [tiab] OR HbA1c [tiab] OR "HbA 1c" [tiab] OR "hemoglobin A1c" [tiab] OR "haemoglobin A1c" [tiab] OR "Hb A1c" [tiab] OR "Hb A1" [tiab] OR hyperglycemia [tiab] OR hyperglycaemia [tiab] OR hyperinsulinemia [tiab] OR hyperinsulinaemia [tiab] OR "blood sugar" [tiab] | [mh "Diabetes mellitus, type 2"] OR [mh "glycated hemoglobin A"] OR [mh Glucose] OR [mh "Insulin Resistance"] OR diabet*:ti,ab OR "glycated haemoglobin A":ti,ab OR "glycated hemoglobin A":ti,ab OR "glycosylated haemoglobin A":ti,ab OR "glycosylated hemoglobin A":ti,ab OR glucose:ti,ab OR glycemic:ti,ab OR glycaemic:ti,ab OR "insulin sensitivity":ti,ab OR "insulin resistance":ti,ab OR prediabetes:ti,ab OR pre-diabetes:ti,ab OR prediabetic:ti,ab OR pre-diabetic:ti,ab OR NIDDM:ti,ab OR T2DM:ti,ab OR T2D:ti,ab OR HbA1:ti,ab OR HbA1c:ti,ab OR "HbA 1c":ti,ab OR "hemoglobin A1c":ti,ab OR "haemoglobin A1c":ti,ab OR "Hb A1c":ti,ab OR "Hb A1":ti,ab OR hyperglycemia:ti,ab OR hyperglycaemia:ti,ab OR hyperinsulinemia:ti,ab OR hyperinsulinaemia:ti,ab OR "blood sugar":ti,ab | 'non insulin dependent diabetes mellitus'/exp OR 'hemoglobin A1c'/exp OR glucose/exp OR 'insulin resistance'/exp OR diabet*:ti,ab OR 'glycated haemoglobin A':ti,ab OR 'glycated hemoglobin A':ti,ab OR 'glycosylated haemoglobin A':ti,ab OR 'glycosylated hemoglobin A':ti,ab OR glucose:ti,ab OR glycemic:ti,ab OR glycaemic:ti,ab OR 'insulin sensitivity':ti,ab OR 'insulin resistance':ti,ab OR prediabetes:ti,ab OR pre-diabetes:ti,ab OR prediabetic:ti,ab OR pre-diabetic:ti,ab OR NIDDM:ti,ab OR T2DM:ti,ab OR T2D:ti,ab OR HbA1:ti,ab OR HbA1c:ti,ab OR 'HbA 1c':ti,ab OR 'hemoglobin A1c':ti,ab OR 'haemoglobin A1c':ti,ab OR 'Hb A1c':ti,ab OR 'Hb A1':ti,ab OR hyperglycemia:ti,ab OR hyperglycaemia:ti,ab OR hyperinsulinemia:ti,ab OR hyperinsulinaemia:ti,ab OR 'blood sugar':ti,ab |

/exp, exploded Emtree terms; mh, MeSH terms; [sb], subject search; ti,ab/tiab, title/abstract;

^1^ PubMed has changed the search strategy of its [sb]-filter for identifying systematic reviews in 01/2019. To maintain continuity we used this previous version for all our literature searches: (systematic review [ti] OR meta-analysis [pt] OR meta-analysis [ti] OR systematic literature review [ti] OR this systematic review [tw] OR pooling project [tw] OR (systematic review [tiab] AND review [pt]) OR meta synthesis [ti] OR meta synthesis [ti] OR integrative review [tw] OR integrative research review [tw] OR rapid review [tw] OR consensus development conference [pt] OR practice guideline [pt] OR drug class reviews [ti] OR cochrane database syst rev [ta] OR acp journal club [ta] OR health technol assess [ta] OR evid rep technol assess summ [ta] OR jbi database system rev implement rep [ta]) OR (clinical guideline [tw] AND management [tw]) OR ((evidence based[ti] OR evidence-based medicine [mh] OR best practice* [ti] OR evidence synthesis [tiab]) AND (review [pt] OR diseases category[mh] OR behavior and behavior mechanisms [mh] OR therapeutics [mh] OR "evaluation studies" [pt] OR “validation studies” [pt] OR guideline [pt] OR pmcbook)) OR ((systematic [tw] OR systematically [tw] OR critical [tiab] OR (study selection [tw]) OR (predetermined [tw] OR inclusion [tw] AND criteri* [tw]) OR exclusion criteri* [tw] OR main outcome measures [tw] OR standard of care [tw] OR standards of care [tw]) AND (survey [tiab] OR surveys [tiab] OR overview* [tw] OR review [tiab] OR reviews [tiab] OR search* [tw] OR handsearch [tw] OR analysis [ti] OR critique [tiab] OR appraisal [tw] OR (reduction [tw] AND (risk [mh] OR risk [tw]) AND (death OR recurrence))) AND (literature [tiab] OR articles [tiab] OR publications [tiab] OR publication [tiab] OR bibliography [tiab] OR bibliographies [tiab] OR published [tiab] OR pooled data [tw] OR unpublished [tw] OR citation [tw] OR citations [tw] OR database [tiab] OR internet [tiab] OR textbooks [tiab] OR references [tw] OR scales [tw] OR papers [tw] OR datasets [tw] OR trials [tiab] OR meta-analy* [tw] OR (clinical [tiab] AND studies [tiab]) OR treatment outcome [mh] OR treatment outcome [tw] OR pmcbook)) NOT (letter [pt] OR newspaper article [pt])
