# Supplementary material for: Protein intake and type 2 diabetes mellitus: an umbrella review of systematic reviews for the evidence-based guideline for protein intake of the German Nutrition Society
Source: Eur J Nutr. 2023 Sep 17;63(1):33–50. doi: 10.1007/s00394-023-03234-5 (PMC10799123; doi:10.1007/s00394-023-03234-5)
Supplement: Supplementary file 2 — Supplementary file2 (DOCX 20 KB) [file 394_2023_3234_MOESM2_ESM.docx]

**Supplementary Material S2** PECO(ST) scheme respectively, for the definition of study eligibility criteria for literature selection.

|  | Category | Inclusion criteria | Exclusion criteria |
| --- | --- | --- | --- |
| P | Population | - General adult population   (≥ 18 years)   - including older adults and recreational athletes - Including people with overweight, obesity, (pre)hypertension and abnormal blood lipids | - Infants, children, adolescents - Pregnant or breastfeeding women - Top athletes |
| E | Exposure^a^ | - Higher protein intake - Intake of total protein - Intake of plant protein - Intake of animal protein - Intake of protein supplements | - Protein was not specifically investigated (e.g. whole food approaches) - Peptides and/or amino acids |
| C | Comparator | - Lower protein intake - Other type of protein intake - Placebo |  |
| O | Outcome^b c^ | - Incidence of type 2 diabetes mellitus |  |
| S | Study design | - SR with or without MA of prospective studies (RCTs or prospective cohort studies, [including nested case-cohort or case-control studies])^b^ | - Individual studies: RCTs, prospective cohort studies, other primary studies - SR of only case-control studies or cross-sectional studies, case studies - Umbrella reviews |
| T | Time | - Any study duration |  |

^a^ The categories will be used as defined by the authors of the systematic review.

^b^ Case-control studies are tolerated if prospective studies are predominant (> 50% of all studies) in the respective systematic review.
