# Supplementary material for: Protein intake and type 2 diabetes mellitus: an umbrella review of systematic reviews for the evidence-based guideline for protein intake of the German Nutrition Society
Source: Eur J Nutr. 2023 Sep 17;63(1):33–50. doi: 10.1007/s00394-023-03234-5 (PMC10799123; doi:10.1007/s00394-023-03234-5)
Supplement: Supplementary file 5 — Supplementary file5 (DOCX 16 KB) [file 394_2023_3234_MOESM5_ESM.docx]

**Supplementary Material S5** Grading the overall certainty of evidence according to methodological quality, outcome-specific certainty of evidence, biological plausibility and consistency of results, and definition of the overall certainty of evidence in a modified form according to the GRADE approach.

| Overall certainty of evidence | Underlying criteria | Definition/Explanation |
| --- | --- | --- |
| Convincing | - At least one SR with MA of prospective studies available (SR without MA only is not sufficient) - If more than one SR with or without MA are available: all overall results must be consistent^1^ - In case of a positive or negative association, biological plausibility is given - All included SRs with MA must reach at least a “moderate” outcome-specific certainty of evidence^2^; in addition, all included SRs must reach at least a methodological quality^3^ of “moderate” | There is high level of confidence that the true effect lies close to that of the estimate(s) of the effect. |
| Probable | - At least one SR with MA of prospective studies available (SR without MA only not sufficient) - If more than one SR with or without MA are available, the majority of overall results must be consistent^1^ - In case of a positive or negative association, biological plausibility is given - The majority^4^ of included SRs with MA must have reached at least a “moderate” outcome-specific certainty of evidence^2^; in addition, all included SRs must reach at least a methodological quality^3^ of “moderate” | There is moderate confidence in the effect estimate(s):  The true effect is likely to be close to the estimate of the effect, but there is a possibility that it is substantially different. |
| Possible | - At least one SR of prospective studies available - If more than one SR with or without MA are available, the majority of overall results must be consistent^1^ - In case of a positive or negative association, biological plausibility is given - The majority^4^ of included SRs with MA must reach at least a “low” outcome-specific certainty of evidence^2^; in addition, the majority^4^ of all included SRs must reach at least a methodological quality^3^ of “moderate” | Confidence in the effect estimate(s) is limited:  The true effect may be substantially different from the estimate of the effect. |
| Insufficient | - No SR available   *OR*   - The majority^4^ of included SRs with MA reach a “very low” outcome-specific certainty of evidence^2^; in addition, the majority of all included SRs reach a methodological quality^3^ of “low” | There is very little confidence in the effect estimate (s):  The true effect is likely to be substantially different from the estimate of effect. |

^1^ Consistent = overall results of the SR have to be consistently either risk reducing or risk elevating or consistently showing no risk association

^2^ Outcome-specific certainty of evidence refers to the NutriGrade rating

^3^ Methodological quality refers the AMSTAR 2 rating**;** SRs graded as “critically low” by AMSTAR 2 are not considered.

^4^ Majority: > 50 % of the included SRs

Abbreviations: MA: meta-analysis; SR: systematic review
