# Supplementary material for: Protein intake and type 2 diabetes mellitus: an umbrella review of systematic reviews for the evidence-based guideline for protein intake of the German Nutrition Society
Source: Eur J Nutr. 2023 Sep 17;63(1):33–50. doi: 10.1007/s00394-023-03234-5 (PMC10799123; doi:10.1007/s00394-023-03234-5)
Supplement: Supplementary file 6 — Supplementary file6 (DOCX 30 KB) [file 394_2023_3234_MOESM6_ESM.docx]

**Supplementary Material S6** List of excluded publications including justifications for exclusion.

| **Reference** | **Reason for exclusion** |
| --- | --- |
| Ajala O, English P, Pinkney J: Systematic review and meta-analysis of different dietary approaches to the management of type 2 diabetes. Am J Clin Nutr 2013;97:505–516. | irrelevant outcome |
| Alvarez-Bueno C, Cavero-Redondo I, Martinez-Vizcaino V, Sotos-Prieto M, Ruiz JR, Gil A: Effects of milk and dairy product consumption on type 2 diabetes: overview of systematic reviews and meta-analyses. Adv Nutr 2019;10, Suppl 2:S154-S163. | relevant diet-disease relationship not investigated |
| Amirani E, Milajerdi A, Reiner Ž, Mirzaei H, Mansournia MA, Asemi Z: Effects of whey protein on glycemic control and serum lipoproteins in patients with metabolic syndrome and related conditions: a systematic review and meta-analysis of randomized controlled clinical trials. Lipids Health Dis 2020;19:209. | irrelevant outcome |
| Anne Fernandez M, Picard-Deland É, Daniel N, Marette A: Yogurt and health: overview of recent data. Cahiers de Nutrition et de Diététique 2017;52, Suppl:S48-S57. | irrelevant language |
| Aune D, Norat T, Romundstad P, Vatten LJ: Dairy products and the risk of type 2 diabetes: a systematic review and dose-response meta-analysis of cohort studies. Am J Clin Nutr 2013;98:1066–1083. | whole food approach |
| Babio N, Bulló M, Salas-Salvadó J: Mediterranean diet and metabolic syndrome: the evidence. Public Health Nutr 2009;12:1607–1617. | irrelevant study type |
| Badely M, Sepandi M, Samadi M, Parastouei K, Taghdir M: The effect of whey protein on the components of metabolic syndrome in overweight and obese individuals; a systematic review and meta-analysis. Diabetes Metab Syndr 2019;13:3121–3131. | irrelevant outcome |
| Becerra-Tomás N, Paz-Graniel I, Hernández-Alonso P, Jenkins DJA, Kendall CWC, Sievenpiper JL, Salas-Salvadó J: Nut consumption and type 2 diabetes risk: a systematic review and meta-analysis of observational studies. Am J Clin Nutr 2021;113:960–971. | relevant diet-disease relationship not investigated |
| Bielefeld D, Grafenauer S, Rangan A: The effects of legume consumption on markers of glycaemic control in individuals with and without diabetes mellitus: a systematic literature review of randomised controlled trials. Nutrients 2020;12. | relevant diet-disease relationship not investigated |
| Blair M, Kellow NJ, Dordevic AL, Evans S, Caissutti J, McCaffrey TA: Health benefits of whey or colostrum supplementation in adults ≥35 years; a systematic review. Nutrients 2020;12. | irrelevant outcome |
| Chalvon-Demersay T, Azzout-Marniche D, Arfsten J, Egli L, Gaudichon C, Karagounis LG, Tome D: A systematic review of the effects of plant compared with animal protein sources on features of metabolic syndrome. J Nutr 2017;147:281–292. | irrelevant outcome |
| Chen M, Sun Q, Giovannucci E, Mozaffarian D, Manson JE, Willett WC, Hu FB: Dairy consumption and risk of type 2 diabetes: 3 cohorts of US adults and an updated meta-analysis. BMC Med 2014;12:215. | whole food approach |
| Chen G-C, Szeto IMY, Chen L-H, Han S-F, Li Y-J, van Hekezen R, Qin L-Q: Dairy products consumption and metabolic syndrome in adults: systematic review and meta-analysis of observational studies. Sci Rep 2015;5:14606. | whole food approach |
| Chen G-C, Zhang Z, van Dam RM, Qin L-Q: Nonlinear relation between animal protein intake and risk of type 2 diabetes. A dose-response meta-analysis of prospective studies. Am J Clin Nutr 2017;105:1014–1016. | irrelevant study type |
| Cherta-Murillo A, Lett AM, Frampton J, Chambers ES, Finnigan TJA, Frost GS: Effects of mycoprotein on glycaemic control and energy intake in humans: a systematic review. Br J Nutr 2020;123:1321–1332. | irrelevant outcome |
| Clifton, P. M., Peters, J., & Keogh, J.: Effect of Dairy Foods on Insulin Sensitivity and Pancreatic Function. Diabetes 2017;66:A204-A204. | only conference abstract available |
| Da Silva MS, Rudkowska I: Dairy products on metabolic health: current research and clinical implications. Maturitas 2014;77:221–228. | irrelevant study type |
| Dämon S, Schätzer M, Höfler J, Tomasec G, Hoppichler F: Nutrition and diabetes mellitus: An overview of the current evidence. Wien Med Wochenschr 2011;161:282–288. | irrelevant study type |
| Dong J-Y, Zhang Z-L, Wang P-Y, Qin L-Q: Effects of high-protein diets on body weight, glycaemic control, blood lipids and blood pressure in type 2 diabetes: meta-analysis of randomised controlled trials. Br J Nutr 2013;110:781–789. | irrelevant population |
| Drouin-Chartier J-P, Li Y, Ardisson Korat AV, Ding M, Lamarche B, Manson JE, Rimm E, Willett WC, Hu F: Changes in Dairy Product Consumption and Risk of Type 2 Diabetes among U.S. Men and Women. Diabetes 2019;68:159-OR. | irrelevant study type |
| Ekmekcioglu C, Wallner P, Kundi M, Weisz U, Haas W, Hutter H-P: Red meat, diseases, and healthy alternatives: a critical review. Crit Rev Food Sci Nutr 2018;58:247–261. | irrelevant study type |
| Fan M, Li Y, Wang C, Mao Z, Zhang L, Yang X, Cui S, Li L: Consumption of Dairy Products in Relation to Type 2 Diabetes Mellitus in Chinese People: The Henan Rural Cohort Study and an Updated Meta-Analysis. Nutrients 2020;12. | relevant diet-disease relationship not investigated |
| Fernandez MA, Panahi S, Daniel N, Tremblay A, Marette A: Yogurt and cardiometabolic diseases: a critical review of potential mechanisms. Adv Nutr 2017;8:812–829. | irrelevant study type |
| Ferreira H, Vasconcelos M, Gil AM, Pinto E: Benefits of pulse consumption on metabolism and health: a systematic review of randomized controlled trials. Crit Rev Food Sci Nutr 2021;61:1–12. | relevant diet-disease relationship not investigated |
| Feskens EJM, Sluik D, van Woudenbergh GJ: Meat consumption, diabetes, and its complications. Curr Diab Rep (Current diabetes reports) 2013;13:298–306. | irrelevant study type |
| Fisher E, Meidtner K, Angquist L, Holst C, Hansen RD, Halkjær J, Masala G, Ostergaard JN, Overvad K, Palli D, Vimaleswaran KS, Tjønneland A, van der A DL, Wareham NJ, Sørensen TI, Loos RJ, Boeing H: Influence of dietary protein intake and glycemic index on the association between TCF7L2 HapA and weight gain 2012;95:1468–1476. | irrelevant study type |
| Franquesa M, Pujol-Busquets G, García-Fernández E, Rico L, Shamirian-Pulido L, Aguilar-Martínez A, Medina FX, Serra-Majem L, Bach-Faig A: Mediterranean diet and cardiodiabesity: a systematic review through evidence-based answers to key clinical questions. Nutrients 2019;11. | irrelevant intervention |
| Gao D, Ning N, Wang C, Wang Y, Li Q, Meng Z, Liu Y, Li Q: Dairy products consumption and risk of type 2 diabetes: systematic review and dose-response meta-analysis. PLoS One 2013;8:e73965. | whole food approach |
| Gijsbers L, Ding EL, Malik VS, Goede J de, Geleijnse JM, Soedamah-Muthu SS: Consumption of dairy foods and diabetes incidence: a dose-response meta-analysis of observational studies. Am J Clin Nutr 2016;103:1111-24. | whole food approach |
| Gil Á, Ortega RM: Introduction and executive summary of the supplement, role of milk and dairy products in health and prevention of noncommunicable chronic diseases: a series of systematic reviews. Adv Nutr 2019;10, Suppl 2:S67-S73. | irrelevant study type |
| Givens DI: Review: dairy foods, red meat and processed meat in the diet: implications for health at key life stages. Animal 2018;12:1709–1721. | irrelevant study type |
| Hengeveld LM, Goede J de, Afman LA, Bakker SJL, Beulens JWJ, Blaak EE, Boersma E, Geleijnse JM, van Goudoever JHB, Hopman MTE, Iestra JA, Kremers SPJ, Mensink RP, Roos NM de, Stehouwer CDA, Verkaik-Kloosterman J, Vet E de, Visser M: Health Effects of Increasing Protein Intake Above the Current Population Reference Intake in Older Adults: A Systematic Review of the Health Council of the Netherlands. Adv Nutr 2021.DOI: 10.1093/advances/nmab140. | Irrelevant outcome |
| Hoffmann, G., & Schwingshackl, L: Systematic review of effects of diets with either low or high glycemic index on cardiovascular and metabolic risk factors. Ann Nutr Metab 2013;63:PO1876. | only conference abstract available |
| Hruby A, Jacques PF: Protein intake and human health: implications of units of protein intake. Adv Nutr 2021;12:71-88 | irrelevant study type |
| Ibsen DB, Steur M, Imamura F, Overvad K, Schulze MB, Bendinelli B, Guevara M, Agudo A, Amiano P, Aune D, Barricarte A, Ericson U, Fagherazzi G, Franks PW, Freisling H, Quiros JR, Grioni S, Heath AK, Huybrechts I, Katze V, Laouali N, Mancini F, Masala G, Olsen A, Papier K, Ramne S, Rolandsson O, Sacerdote C, Sánchez M-J, Santiuste C, Simeon V, Spijkerman AMW, Srour B, Tjønneland A, Tong TYN, Tumino R, van der Schouw YT, Weiderpass E, Wittenbecher C, Sharp SJ, Riboli E, Forouhi NG, Wareham NJ: Replacement of red and processed meat with other food sources of protein and the risk of type 2 diabetes in European populations: the EPIC-InterAct Study. Diabetes Care 2020;43:2660–2667. | irrelevant study type |
| Jamioł-Milc D, Biernawska J, Liput M, Stachowska L, Domiszewski Z: Seafood Intake as a Method of Non-Communicable Diseases (NCD) Prevention in Adults. Nutrients 2021;13. | irrelevant study type |
| Jannasch F, Kröger J, Schulze MB: Dietary patterns and type 2 diabetes: a systematic literature review and meta-analysis of prospective studies. J Nutr 2017;147:1174–1182. | irrelevant intervention |
| Kendall, C. W., Sievenpiper, J. L., Esfahani, A., Wong, J. M., Carleton, A. J., Jiang, H. Y: Legumes and glycemic control: a meta-analysis of randomized controlled trials. Eur Heart J 2009;30:584. | only conference abstract available |
| Khoramdad M, Alimohamadi Y, Safiri S, Pakzad R, Shakiba E, Shafiei J, Firouzi A: Dairy products consumption and risk of type 2 diabetes: a systematic review and meta-analysis of prospective cohort studies. Iran Red Crescent Med J 2017;19. | whole food approach |
| Khoramdad M, Rahimi M, Cheraghi Z, Izadi N, Alimohamadi Y, Firouzi A, Shafiei J: The effect of dairy products subgroups consumption on the risk of diabetes: a systematic review and meta-analysis. Iran Red Crescent Med J 2016;19. | whole food approach |
| Lee M, Lee H, Kim J: Dairy food consumption is associated with a lower risk of the metabolic syndrome and its components: a systematic review and meta-analysis. Br J Nutr 2018;120:373–384. | whole food approach |
| Livesey G, Taylor R, Livesey HF, Buyken AE, Jenkins DJA, Augustin LSA, Sievenpiper JL, Barclay AW, Liu S, Wolever TMS, Willett WC, Brighenti F, Salas-Salvadó J, Björck I, Rizkalla SW, Riccardi G, La Vecchia C, Ceriello A, Trichopoulou A, Poli A, Astrup A, Kendall CWC, Ha M-A, Baer-Sinnott S, Brand-Miller JC: Dietary glycemic index and load and the risk of type 2 diabetes: a systematic review and updated meta-analyses of prospective cohort studies. Nutrients 2019;11. | irrelevant exposure |
| Lombardo M, Bellia C, Moletto C, Aulisa G, Padua E, Della-Morte D, Caprio M, Bellia A: Effects of quality and quantity of protein intake for type 2 diabetes mellitus prevention and metabolic control. Curr Nutr Rep 2020;9:329–337. | irrelevant study type |
| Lonnie M, Laurie I, Myers M, Horgan G, Russell WR, Johnstone AM: Exploring health-promoting attributes of plant proteins as a functional ingredient for the food sector: a systematic review of human interventional studies. Nutrients 2020;12. | irrelevant outcome |
| Martini LA, Wood RJ: Milk intake and the risk of type 2 diabetes mellitus, hypertension and prostate cancer. Arq Bras Endocrinol Metabol 2009;53:688–694. | irrelevant study type |
| McEvoy CT, Temple N, Woodside JV: Vegetarian diets, low-meat diets and health: a review. Public Health Nutr 2012;15:2287–2294. | irrelevant intervention |
| Meng H, Matthan NR, Ausman LM, Lichtenstein AH: Effect of macronutrients and fiber on postprandial glycemic responses and meal glycemic index and glycemic load value determinations. Am J Clin Nutr 2017;105:842–853. | irrelevant study type |
| Miller V, Micha R, Choi E, Karageorgou D, Webb P, Mozaffarian D: Evaluation of the quality of evidence of the association of foods and nutrients with cardiovascular disease and diabetes: a systematic review. JAMA Netw Open 2022;5:e2146705. | irrelevant study type |
| Mishali M, Prizant-Passal S, Avrech T, Shoenfeld Y: Association between dairy intake and the risk of contracting type 2 diabetes and cardiovascular diseases: a systematic review and meta-analysis with subgroup analysis of men versus women. Nutr Rev 2019;77:417–429. | relevant diet-disease relationship not investigated |
| Mohammadifard N, Sajjadi F, Haghighatdoost F: Effects of soy consumption on metabolic parameters in patients with metabolic syndrome: A systematic review and meta-analysis. EXCLI J 2021;20:665–685. | irrelevant outcome |
| Nagata C. Soy intake and chronic disease risk: findings from prospective cohort studies in Japan. Eur J Clin Nutr. 2021 Jun;75(6):890-901. doi: 10.1038/s41430-020-00744-x. | irrelevant exposure |
| Nordmann AJ, Suter-Zimmermann K, Bucher HC, Shai I, Tuttle KR, Estruch R, Briel M: Meta-analysis comparing Mediterranean to low-fat diets for modification of cardiovascular risk factors. Am J Med 2011;124:841. | irrelevant intervention |
| Ntzouvani A, Antonopoulou S, Nomikos T: Effects of nut and seed consumption on markers of glucose metabolism in adults with prediabetes: A systematic review of randomised controlled trials. Br J Nutr 2019;122:361–375. | relevant diet-disease relationship not investigated |
| O'Connor LE, Kim JE, Clark CM, Zhu W, Campbell WW: Effects of total red meat intake on glycemic control and inflammatory biomarkers: a meta-analysis of randomized controlled trials. Adv Nutr 2021;12:115–127. | relevant diet-disease relationship not investigated |
| O'Connor S, Turcotte A-F, Gagnon C, Rudkowska I: Increased dairy product intake modifies plasma glucose concentrations and glycated hemoglobin: a systematic review and meta-analysis of randomized controlled trials. Adv Nutr 2019;10:262–279. | relevant diet-disease relationship not investigated |
| Pan A, Sun Q, Bernstein AM, Schulze MB, Manson JE, Willett WC, Hu FB: Red meat consumption and risk of type 2 diabetes: 3 cohorts of US adults and an updated meta-analysis. Am J Clin Nutr 2011;94:1088–1096. | whole food approach |
| Papadaki A, Nolen-Doerr E, Mantzoros CS: The effect of the mediterranean diet on metabolic health: a systematic review and meta-analysis of controlled trials in adults. Nutrients 2020;12. | relevant diet-disease relationship not investigated |
| Pasin G, Comerford KB: Dairy foods and dairy proteins in the management of type 2 diabetes: a systematic review of the clinical evidence. Adv Nutr 2015;6:245–259. | irrelevant population |
| Pearce M, Fanidi A, Bishop TRP, Sharp SJ, Imamura F, Dietrich S, Akbaraly T, Bes-Rastrollo M, Beulens JWJ, Byberg L, Canhada S, Del Molina MCB, Chen Z, Cortes-Valencia A, Du H, Duncan BB, Härkänen T, Hashemian M, Kim J, Kim MK, Kim Y, Knekt P, Kromhout D, Lassale C, Ridaura RL, Magliano DJ, Malekzadeh R, Marques-Vidal P, Martínez-González MÁ, O'Donoghue G, O'Gorman D, Shaw JE, Soedamah-Muthu SS, Stern D, Wolk A, Woo HW, Wareham NJ, Forouhi NG: Associations of Total Legume, Pulse, and Soy Consumption with Incident Type 2 Diabetes: Federated Meta-Analysis of 27 Studies from Diverse World Regions. J Nutr 2021;151:1231–1240. | whole food approach |
| Psaltopoulou T, Ilias I, Alevizaki M: The role of diet and lifestyle in primary, secondary, and tertiary diabetes prevention: a review of meta-analyses. Rev Diabet.Stud 2010;7:26–35. | irrelevant study type |
| Richard C, Cristall L, Fleming E, Lewis ED, Ricupero M, Jacobs RL, Field CJ: Impact of egg consumption on cardiovascular risk factors in individuals with type 2 diabetes and at risk for developing diabetes. A systematic review of randomized nutritional intervention studies. Can J Diabetes 2017;41:453-463. | whole food approach |
| Salari A, Ghodrat S, Gheflati A, Jarahi L, Hashemi M, Afshari A: Effect of kefir beverage consumption on glycemic control: a systematic review and meta-analysis of randomized controlled clinical trials. Complement Ther Clin Pract 2021;44:101443. | Irrelevant exposure |
| Salas-Salvadó J, Guasch-Ferré M, Díaz-López A, Babio N: Yogurt and diabetes: overview of recent observational studies. J Nutr 2017;147, Suppl:1452S-1461S. | irrelevant study type |
| Santesso N, Akl EA, Bianchi M, Mente A, Mustafa R, Heels-Ansdell D, Schünemann HJ: Effects of higher- versus lower-protein diets on health outcomes: a systematic review and meta-analysis. Eur J Clin Nutr 2012;66:780–788. | irrelevant outcome |
| Savaiano DA, Hutkins RW: Yogurt, cultured fermented milk, and health: a systematic review. Nutr Rev DOI: 10.1093/nutrit/nuaa013. | relevant diet-disease relationship not investigated |
| Schwingshackl L, Hoffmann G: Long-term effects of low-fat diets either low or high in protein on cardiovascular and metabolic risk factors: a systematic review and meta-analysis. Nutr J 2013;12:48. | irrelevant outcome |
| Schwingshackl L, Hoffmann G, Lampousi A-M, Knüppel S, Iqbal K, Schwedhelm C, Bechthold A, Schlesinger S, Boeing H: Food groups and risk of type 2 diabetes mellitus: a systematic review and meta-analysis of prospective studies. Eur J Epidemiol 2017;32:363–375. | whole food approach |
| Schwingshackl L, Hoffmann G, Iqbal K, Schwedhelm C, Boeing H: Food groups and intermediate disease markers: a systematic review and network meta-analysis of randomized trials. Am J Clin Nutr 2018;108:576–586. | irrelevant study type |
| Schwingshackl L, Knüppel S, Michels N, Schwedhelm C, Hoffmann G, Iqbal K, de Henauw S, Boeing H, Devleesschauwer B: Intake of 12 food groups and disability-adjusted life years from coronary heart disease, stroke, type 2 diabetes, and colorectal cancer in 16 European countries. Eur J Epidemiol 2019;34:765–775. | relevant diet-disease relationship not investigated |
| Shah M, Garg A: The relationships between macronutrient and micronutrient intakes and type 2 diabetes mellitus in South Asians: a review. J Diabetes Complicat 2019;33:500–507. | irrelevant study type |
| Sluijs I, Forouhi NG, Beulens JWJ, van der Schouw YT, Agnoli C, Arriola L, Balkau B, Barricarte A, Boeing H, Bueno-de-Mesquita HB, Clavel-Chapelon F, Crowe FL, Lauzon-Guillain B de, Drogan D, Franks PW, Gavrila D, Gonzalez C, Halkjaer J, Kaaks R, Moskal A, Nilsson P, Overvad K, Palli D, Panico S, Quirós JR, Ricceri F, Rinaldi S, Rolandsson O, Sacerdote C, Sánchez M-J, Slimani N, Spijkerman AMW, Teucher B, Tjonneland A, Tormo M-J, Tumino R, van der A DL, Sharp SJ, Langenberg C, Feskens EJM, Riboli E, Wareham NJ: The amount and type of dairy product intake and incident type 2 diabetes: results from the EPIC-InterAct Study. Am J Clin Nutr 2012;96:382–390. | irrelevant study type |
| Sluik D, Brouwer-Brolsma EM, Berendsen AAM, Mikkilä V, Poppitt SD, Silvestre MP, Tremblay A, Pérusse L, Bouchard C, Raben A, Feskens EJM: Protein intake and the incidence of pre-diabetes and diabetes in 4 population-based studies: the PREVIEW project. Am J Clin Nutr 2019;109:1310–1318. | irrelevant study type |
| Sochol KM, Johns TS, Buttar RS, Randhawa L, Sanchez E, Gal M, Lestrade K, Merzkani M, Abramowitz MK, Mossavar-Rahmani Y, Melamed ML: The effects of dairy intake on insulin resistance: a systematic review and meta-analysis of randomized clinical trials. Nutrients 2019;11:2237. | irrelevant population |
| Soedamah-Muthu SS, Goede J de: Dairy consumption and cardiometabolic diseases: systematic review and updated meta-analyses of prospective cohort studies. Curr Nutr Rep 2018;7:171–182. | whole food approach |
| Stelmach-Mardas M, Walkowiak J: Dietary interventions and changes in cardio-metabolic parameters in metabolically healthy obese subjects: a systematic review with meta-analysis. Nutrients 2016;8. | irrelevant intervention |
| Thorning TK, Raben A, Tholstrup T, Soedamah-Muthu SS, Givens I, Astrup A: Milk and dairy products: good or bad for human health? An assessment of the totality of scientific evidence. Food Nutr Res 2016;60:32527. | irrelevant study type |
| Tong X, Dong J-Y, Wu Z-W, Li W, Qin L-Q: Dairy consumption and risk of type 2 diabetes mellitus: a meta-analysis of cohort studies. Eur J Clin Nutr 2011;65:1027–1031. | whole food approach |
| Turner KM, Keogh JB, Clifton PM: Dairy consumption and insulin sensitivity: a systematic review of short- and long-term intervention studies. Nutr Metab Cardiovasc Dis 2015;25:3–8. | irrelevant outcome |
| Ventriglio A, Sancassiani F, Contu MP, Latorre M, Di Slavatore M, Fornaro M, Bhugra D: Mediterranean Diet and its Benefits on Health and Mental Health: A Literature Review. Clin Pract Epidemiol Ment Health 2020;16:156–164. | relevant diet-disease relationship not investigated |
| Vogtschmidt YD, Raben A, Faber I, Wilde C de, Lovegrove JA, Givens DI, Pfeiffer AFH, Soedamah-Muthu SS: Is protein the forgotten ingredient: effects of higher compared to lower protein diets on cardiometabolic risk factors. A systematic review and meta-analysis of randomised controlled trials. Atherosclerosis 2021;328:124–135. | irrelevant outcome |
| Walker KZ, O'Dea K, Gomez M, Girgis S, Colagiuri R: Diet and exercise in the prevention of diabetes. J Hum Nutr Diet 2010;23:344–352. | relevant diet-disease relationship not investigated |
| White DL, Collinson A: Red meat, dietary heme iron, and risk of type 2 diabetes: the involvement of advanced lipoxidation endproducts. Adv Nutr 2013;4:403–411. | whole food approach |
| Yang B, Chen Y, Xu T, Yu Y, Huang T, Hu X, Li D: Systematic review and meta-analysis of soy products consumption in patients with type 2 diabetes mellitus. Asia Pac J Clin Nutr 2011;20:593–602. | irrelevant population |
| Yu E, Hu FB: Dairy products, dairy fatty acids, and the prevention of cardiometabolic disease: a review of recent evidence. Curr Atheroscler Rep 2018;20:24. | irrelevant study type |
| Yuan S, Larsson SC: An atlas on risk factors for type 2 diabetes: a wide-angled Mendelian randomisation study. Diabetologia 2020;63:2359–2371. | irrelevant study type |
| Zhang X-M, Zhang Y-B, Chi M-H: Soy protein supplementation reduces clinical indices in type 2 diabetes and metabolic syndrome. Yonsei Med J 2016;57:681–689. | irrelevant population |
