# Supplementary material for: Protein intake and type 2 diabetes mellitus: an umbrella review of systematic reviews for the evidence-based guideline for protein intake of the German Nutrition Society
Source: Eur J Nutr. 2023 Sep 17;63(1):33–50. doi: 10.1007/s00394-023-03234-5 (PMC10799123; doi:10.1007/s00394-023-03234-5)
Supplement: Supplementary file 7 — Supplementary file7 (DOCX 18 KB) [file 394_2023_3234_MOESM7_ESM.docx]

**Supplementary Material S7** Overview of all primary studies included in the SRs with or without meta-analysis on dietary protein intake and type 2 diabetes risk.

|  |  |  |  |  |  |  |  |  |  |  |
| --- | --- | --- | --- | --- | --- | --- | --- | --- | --- | --- |
| **First Author** | **Publication Year** | **Study** | **Boushey**  **2020** | **Fan**  **2019** | **Ye 2019** | **Zhao 2018** | **Tian 2017** | **Shang 2016** | **Pedersen**  **2013** | **Alhazmi  2012** |
| Chen | 2020 [41] | Rotterdam Study | x | x |  |  |  |  |  |  |
| Virtanen | 2017 [39] | Kuopio Ischaemic Heart Disease Risk Factor Study | x | x | x | x | x |  |  |  |
| Shang | 2016 [20] | Melboune Collaborative Cohort | x | x | x | x | x | x |  |  |
| Malik | 2016 [40] | Nurses’ Health Study | x | x | x | x | x | x |  |  |
| Malik | 2016 [40] | Nurses’ Health Study II | x | x | x | x | x | x |  |  |
| Malik | 2016 [40] | Health Professionals Follow-up Study | x | x | x | x | x | x |  |  |
| Nanri | 2015 [38] | Japan Public Health Center-Based Prospective Study | x |  | x | x | x | x |  |  |
| van Nielen | 2014 [37] | European Prospective Investigation into Cancer and Nutrition | x |  | x | x | x | x |  |  |
| Alhazmi | 2014 [36] | Australian Longitudinal Study on Women’s Health |  |  | x | x |  | x |  |  |
| Ericson | 2013 [35] | Malmo Diet and Cancer cohort | x | x | x |  | x | x |  |  |
| Bao | 2013 [34] | Nurses’ Health Study II* |  |  |  |  | x |  |  |  |
| Tinker | 2011 [33] | Women’s Health Initiative |  |  | x | x | x | x |  |  |
| Sluijs | 2010 [32] | European Prospective Investigation into Cancer and Nutrition- NL | x | x | x |  | x | x | x | x |
| de Koning | 2011 [31] | Health Professionals Follow-Up Study | x |  |  |  |  |  | x |  |
| Schulze | 2008 [30] | European Prospective Investigation into Cancer and Nutrition -Potsdam Study | x |  |  |  |  |  | x |  |
| Halton | 2008 [29] | Nurses’ Health Study |  |  |  |  |  |  | x | x |
| Song | 2004 [28] | Women’s Health Study |  |  | x | x |  |  |  |  |
| Colditz | 1992 [27] | Nurses’ Health Study |  |  |  |  |  |  |  | x |

* study outcome was gestational diabetes
