# Supplementary material for: Protein intake and type 2 diabetes mellitus: an umbrella review of systematic reviews for the evidence-based guideline for protein intake of the German Nutrition Society
Source: Eur J Nutr. 2023 Sep 17;63(1):33–50. doi: 10.1007/s00394-023-03234-5 (PMC10799123; doi:10.1007/s00394-023-03234-5)
Supplement: Supplementary file 8 — Supplementary file8 (DOCX 18 KB) [file 394_2023_3234_MOESM8_ESM.docx]

**Supplementary Material S8** Methodological quality assessment of systematic reviews using AMSTAR 2.

| Assessment  item  Systematic review | 1. Components of PICO included? | 1. Method a priori? Protocol reported? | 1. Comprehensive literature search strategy? | 1. Study selection performed in duplicate? | 1. Data extraction performed in duplicate? | 1. Number of excluded studies and corresponding reasons provided? | 1. Detailed study characteristics provided? | 1. Risk of bias assessed? | 1. Statistical heterogeneity assessed? | 1. Risk of bias considered in the discussion and interpretation? | 1. Discussion of any heterogeneity observed in the results? | 1. Publication bias investigated? | 1. Likely impact of existing publication bias discussed? | 1. Potential conflicts of interest stated? | Number of critical weaknesses | Number of non-critical weaknesses | Methodological quality |
| --- | --- | --- | --- | --- | --- | --- | --- | --- | --- | --- | --- | --- | --- | --- | --- | --- | --- |
| Boushey 2020 [24] | Yes | Yes | Yes | Yes | Yes | Yes | Yes | Yes | No MA | Yes | No MA | No MA | No MA | Yes | 0 | 0 | high |
| Fan, 2019 [23] | No | No | Yes | No | Yes | Yes | Yes | Yes | Yes | Yes | Yes | Yes | Yes | Yes | 0 | 3 | moderate |
| Ye 2019 [25] | Yes | Yes | Yes | Yes | Yes | Yes | Yes | Yes | Yes | Yes | No | Yes | Yes | Yes | 0 | 1 | high |
| Zhao 2018 [22] | Yes | Yes | Yes | Yes | Yes | Yes | Yes | Yes | Yes | No | Yes | Yes | Yes | Yes | 0 | 1 | high |
| Tian 2017 [21] | Yes | No | Yes | No | No | Yes | No | No | Yes | No | Yes | Yes | Yes | Yes | 1 | 5 | low |
| Shang 2016 [20] | Yes | No | Yes | No | No | Yes | Yes | Yes | Yes | Yes | Yes | Yes | Yes | Yes | 0 | 3 | moderate |
| Pedersen 2013 [13] | Yes | Yes | Yes | Yes | No | Yes | Yes | Yes | No MA | No | No MA | No MA | No MA | Yes | 0 | 3 | moderate |
| Alhazmi 2012 [19] | Yes | Yes | Yes | Yes | Yes | No | Yes | Yes | Yes | Yes | Yes | Yes | Yes | Yes | 1 | 0 | low |

The assessment items are provided as shortened versions. The full questionnaire is provided in Supplementary Material S3. Critical assessment items are underlined.

High rating = no critical weakness with no or one non-critical weakness: the systematic review provides an accurate and comprehensive summary of the results of the available studies that address the question of interest. Moderate rating = no critical weakness with more than one non-critical weakness: the systematic review has more than one weakness but no critical flaws. It may provide an accurate summary of the results of the available studies that were included in the review. Low rating = one critical weakness with or without non-critical weaknesses: the review has a critical flaw and may not provide an accurate and comprehensive summary of the available studies that address the question of interest. Critically low rating = more than one critical weakness with or without non-critical weaknesses: the review has more than one critical flaw and should not be relied on to provide an accurate and comprehensive summary of the available studies. Shea BJ, Reeves BC, Wells G, Thuku M, Hamel C, Moran J, et al. AMSTAR 2: a critical appraisal tool for systematic reviews that include randomised or non-randomised studies of healthcare interventions, or both. BMJ. 2017 Sep;358(j4008):1-8. AMSTAR 2: A Measurement Tool to Assess Systematic Reviews 2; MA: meta-analysis
