# Supplementary material for: Protein intake and type 2 diabetes mellitus: an umbrella review of systematic reviews for the evidence-based guideline for protein intake of the German Nutrition Society
Source: Eur J Nutr. 2023 Sep 17;63(1):33–50. doi: 10.1007/s00394-023-03234-5 (PMC10799123; doi:10.1007/s00394-023-03234-5)
Supplement: Supplementary file 9 — Supplementary file9 (DOCX 23 KB) [file 394_2023_3234_MOESM9_ESM.docx]

**Supplementary Material S9** Characteristics of SRs on glycaemic traits.

| **Author,  year** | **Study type & study period** | **Study population** | **Exposition** | **Protein intake** | **Endpoint/outcome** | **Effect estimates** | **Heterogeneity estimators** | **AMSTAR 2 Rating** |
| --- | --- | --- | --- | --- | --- | --- | --- | --- |
| O'Connor, 2021   [41] | SR with MA of RCTs published until 08/2019 | Both sexes age: ≥ 19 yrs | High vs. Low protein |  |  | WMD (95% CI)  random effects model: |  | High |
|  |  |  |  | 8 RCTs | Glucose (mmol/L) | 0.115 (-0.004, 0.235) | I²= 15 % |  |
|  |  |  |  | 5 RCTs | Insulin (pmol/L) | -11.023 (-21.554, -0.492) | I²= 1 % |  |
|  |  |  |  | 6 RCTs | HOMA-IR | -0.332 (-0.685, 0.020) | I²= 0 % |  |
| Mohammadifard, 2021   [42] | SR with MA of RCTs published until 06/2020 | Age: ≥ 18 yrs  Metabolic syndrome |  | soy protein |  | MD (95% CI)  random effects model: |  | Low |
|  |  | 3 RCTs |  |  | FBS | -0.38 (-0.68, -0.09) | I²= 71 % |  |
|  |  | 3 RCTs |  |  | insulin | -0.90 (-1.22, -0.59) | I²= 93 % |  |
|  |  | 3 RCTs |  |  | HOMA-IR | -0.86 (-1.17, -0.55) | I²= 93 % |  |
| Amirani, 2020  [43] | SR with MA of RCTs published until 04/2020 study duration: 4-24 wks | Both sexes age: ≥ 18 yrs mild hypertension, overweight, obese, PCOs, on energy reduction, prehypertension, T2DM, metabolic syndrome, with gastric bypass | Whey protein vs. control | 4.3-90 g/d or 0.4-0.5 g/kg BW |  | WMD (95% CI)  random effects model: |  | High |
|  |  | 20 RCTs  n = 850 participants |  |  | FPG | -0.61 (-2.83, 1.62) | I²= 90 %  p < 0.001 |  |
|  |  | 14 RCTs  n = 691 participants |  |  | insulin | -0.94 (-1.68, -0.21) | I²= 63 %  p < 0.001 |  |
|  |  | 13 RCTs  n = 1,938 participants |  |  | HOMA-IR | -0.20 (-0.36, -0.05) | I²= 67 %  p = 0.000 |  |
|  |  | 6 RCTs  n = 304 participants |  |  | HbA1c | -0.15 (-0.29, -0.01) | I²= 91 %  p < 0.001 |  |
| Blair, 2020  [44] | SR without MA of RCTs  published until 09/2018 study duration: 2-23 wks | Both sexes age: ≥ 35 yrs |  | Whey protein  20-60 g/d |  |  |  | High |
|  |  | 1 RCT  n = 23 participants | Whey protein concentrate |  | FGL | "No changes were reported in FGLs after WPC" | NA |  |
|  |  | 2 RCTs  n = 39 participants | Whey protein isolate |  | FGL | "No changes were reported in FGLs after […] WPI" |  |  |
|  |  | 1 RCT  n = 18 participants | Non-specific whey |  | FGL | "No changes were reported in FGLs after […] non-specific whey supplementation" |  |  |
|  |  | 1 RCT  n = 14 participants | Whey protein isolate |  | Postprandial glucose level | "No changes were reported […] in postprandial glucose levels after WPI" |  |  |
|  |  | 1 RCT  n = 14 participants | Whey protein isolate |  | Postprandial insulin level | "There was no change in postprandial insulin after WPI" |  |  |
|  |  | 1 RCT  n = 18 participants | Non-specific whey |  | FIL | "There was no change in […] fasting insulin after non-specific whey" |  |  |
|  |  | 1 RCT  n = 23 participants | Whey protein concentrate vs. maltodextrin |  | FIL | "Fasting insulin was reduced when compared to […] a maltodextrin […] control" |  |  |
|  |  | 1 RCT  n = 25 participants | Whey protein isolate vs. Glucose |  | FIL | "Fasting insulin was reduced when compared to […] a […] glucose control" |  |  |
|  |  | 1 RCT  n = 23 participants | Whey protein concentrate vs. soy |  | FIL | "Fasting insulin was […] not significantly different when compared to soy protein" |  |  |
|  |  | 1 RCT  n = 25 participants | Whey protein isolate vs. glucose |  | HOMA-IR | "Fasting homeostatic model assessment of insulin resistance (HOMA-IR) was reduced after WPI consumption when compared to a glucose control" |  |  |
|  |  | 1 RCT  n = 18 participants | Whey protein isolate vs.non-specific whey as addition to resistance training |  | HOMA-IR | "Fasting homeostatic model assessment of insulin resistance (HOMA-IR) was unchanged when compared to non-specific whey as addition to resistance training" |  |  |
| Cherta-Murillo,  2020  [45] | SR without MA of RCTs published up to 11/2019 study duration: 120-240 min | Both sexes mean age: 23 - 33 yrs healthy, lean, overweight, obese | Mycoprotein vs. control | Supplement: 20-132 g |  |  | NA | Moderate |
|  |  | 3 RCTs  n = 67 participants |  |  | Insulin AUC, glucose AUC | "In summary, the effects of mycoprotein on glycaemia are unclear. However, the data suggest that the acute intake of mycoprotein may decrease insulin output in healthy lean and overweight adults." |  |  |
| Lonnie, 2020   [46] | SR without MA of RCTs published until 03/2020  study duration: 1 d - 6 wks | Both sexes mean age: 22 - 54 yrs lean, obese | Plant protein vs. control | 10-70 g/d or 0.25-0.6 g/kg BW/d | Glycaemia |  | NA | Low |
|  |  | 18 RCTs  n = 592 participants |  |  |  | "The majority of studies suggested a beneficial effect of extracted plant proteins on regulating postprandial glycemia, in comparison to controls and other protein sources. […] the evidence points towards direction, that investigated plant proteins had a similar, but not a superior effect to that from animal protein (mainly milk proteins [...]). |  |  |
| Badely 2019  [47] | SR with MA of RCTs published between 01/2000 and 05/2019 study duration: 2-36 wks | Both sexes overweight, obese mean age: 23 - 78 yrs | Whey protein vs.placebo | 0.7-100.2 g/d or 1.5-1.7 g/kg BW/d |  | WMD (95% CI): |  | Moderate |
|  |  | 28 RCTs  n = 1,878 participants |  |  | FBS | -1.42 (-1.52, -1.31) | I²= 100 %  p = 0.000 |  |
| Schwingshackl 2013  [12] | MA of RCTs  published before 08/2012 study duration: 12 to 24 mo | Both sexes healthy, overweight, insulin resistant or T2DM  age: 20 - 65 yrs | High protein (≥25En%) vs. Low protein (≤20En%) with both Low fat (≤30En%) | 25-40 EN% vs. 10-20 EN% |  | pooled WMD (95% CI), random effect model: |  | High |
|  |  |  |  |  | fasting insulin (µIU/ml) | -0.71 (-1.36, -0.05) | I²=0 % |  |
|  |  |  |  |  | fasting glucose (mg/dl) | -0.63 (-1.93, 0.67) | I²=0 % |  |
|  |  |  |  |  | HbA1c (%) | 0.07 (-0.17, 0.31) | I²=0 % |  |
| Santesso 2012  [15] | MA of RCTs  published before 08/11  study duration: 28 to 356 d | Both sexes average age: 45 yrs  healthy, overweight, obese, hyperlipidaemia, hypertension or MS | Higher- vs. lower-protein diets (≥ 5% difference in total energy intake) | 16-45 EN% (median: 27 EN%) vs. 5-23 EN% (median: 18 EN%) | - HbA1c - FBG - FBI | MA conducted with change values: |  | High |
|  |  |  |  |  | HbA1c | MD (95% CI)  randon effect model: 0.00 (-0.19, 0.19) | I²=0 % |  |
|  |  |  |  |  | FBG | SMD (95% CI)  random effect model: -0.05 (-0.20, 0.10) | I²=29 % |  |
|  |  |  |  |  | FBI | SMD (95% CI)  random effect model: -0.20 (-0.37, -0.03) | I²=2 3% |  |
|  |  |  |  |  |  | MA conducted with end of study values: |  |  |
|  |  |  |  |  | FBG | SMD (95% CI)  random effect model: 0.06 (-0.05, 0.17) | I²=22 % |  |
|  |  |  |  |  | FBI | SMD (95% CI)  random effect model: -0.26 (-0.59, 0.07) | NP |  |

Abbreviations: AMSTAR 2: A Measurement Tool to Assess Systematic Reviews 2; AUC: area under curve; BMI: body mass index; CI: confidence interval; d: day; En%: energy percentage; FBG: fasting blood glucose; FBI: fasting blood insulin; FBS: fasting blood sugar; FPG: fasting plasma glucose; FGL: fasting glucose level; FIL: fasting insulin level; HbA1c: haemoglobin A1c; HOMA.IR: Homoeostasis Model Assessment-Index; LHCP: Low-carbohydrate-High-protein; MA: meta-analysis; MD: mean difference; min: minute; mo: month; MS: metabolic syndrome; NA: not applicable; NP: not provided; Q: Cochran’s Q; RCT: randomised controlled trial; RR: risk ratio; SMD: standardised mean difference; SR: systematic review; T2D: type 2 diabetes mellitus; wk: week; WMD: weighted mean difference; yrs: years;
